# Expression of SnoRNA U50A Is Associated with Better Prognosis and Prolonged Mitosis in Breast Cancer

**DOI:** 10.3390/cancers13246304

**Published:** 2021-12-15

**Authors:** Jie-Ning Li, Ming-Yang Wang, Yi-Ting Chen, Yao-Lung Kuo, Pai-Sheng Chen

**Affiliations:** 1 Institute of Basic Medical Sciences, College of Medicine, National Cheng Kung University, Tainan 701, Taiwan; z11002049@ncku.edu.tw; 2 Department of Medical Laboratory Science and Biotechnology, College of Medicine, National Cheng Kung University, Tainan 701, Taiwan; i54051199@gs.ncku.edu.tw; 3 Department of Surgery, National Taiwan University Hospital, Taipei 100, Taiwan; A00581@ntucc.gov.tw; 4 Department of Medicine, College of Medicine, National Cheng Kung University, Tainan 701, Taiwan; 5 Department of Surgery, College of Medicine, National Cheng Kung University, Tainan 701, Taiwan; 6 Breast Medical Center, National Cheng Kung University Hospital, Tainan 701, Taiwan

**Keywords:** breast cancer, snoRNA, mitosis, cancer prognosis

## Abstract

**Simple Summary:**

SnoRNAs are essential for fundamental cellular processes. However, emerging evidence shows that snoRNAs play regulatory roles during cancer progression. The snoRNA U50A (U50A) is a newly-identified putative tumor suppressor, but its clinical and mechanistic impacts in breast cancer remain elusive. In this study, we quantified the copy number of U50A in breast cancer patient tissues and found that a higher level of U50A expression is correlated with better overall survival in breast cancer patients. By utilizing transcriptomic analysis, we demonstrated that U50A prolongs mitosis and reduces colony-forming ability through downregulating mitosis-related genes. Consistent with these in vitro results, breast cancer tissues expressing higher U50A significantly exhibited accumulated mitotic tumor cells and were associated with reduced tumor size. Altogether, this is the first study showing the clinical, cellular, and regulatory impacts of snoRNA U50A in human breast cancer.

**Abstract:**

Small nucleolar RNAs (snoRNAs) are small noncoding RNAs generally recognized as housekeeping genes. Genomic analysis has shown that snoRNA U50A (U50A) is a candidate tumor suppressor gene deleted in less than 10% of breast cancer patients. To date, the pathological roles of U50A in cancer, including its clinical significance and its regulatory impact at the molecular level, are not well-defined. Here, we quantified the copy number of U50A in human breast cancer tissues. Our results showed that the U50A expression level is correlated with better prognosis in breast cancer patients. Utilizing RNA-sequencing for transcriptomic analysis, we revealed that U50A downregulates mitosis-related genes leading to arrested cancer cell mitosis and suppressed colony-forming ability. Moreover, in support of the impacts of U50A in prolonging mitosis and inhibiting clonogenic activity, breast cancer tissues with higher U50A expression exhibit accumulated mitotic tumor cells. In conclusion, based on the evidence from U50A-downregulated mitosis-related genes, prolonged mitosis, repressed colony-forming ability, and clinical analyses, we demonstrated molecular insights into the pathological impact of snoRNA U50A in human breast cancer.

## 1. Introduction

Breast cancer is the most common cancer type among women, with an increasing incidence rate worldwide, and is the second leading cause of cancer death [1,2]. For clinical implications and treatment strategies, immunohistochemistry (IHC) markers, such as estrogen receptor (ER), progesterone receptor (PR), and human epidermal growth factor receptor 2 (HER2), combined with clinicopathological variables, including tumor size, tumor grade, and nodal involvement, have been routinely utilized [3,4]. With the progression of preventive medicine, breast cancer can be diagnosed in the early stages. However, there is no suitable prognostic marker for the early prediction of breast cancer survival. Furthermore, traditional pathological markers used to classify breast tissues into TNM stages have limitations for discriminating individual variability. Hence, the need for establishing new molecular diagnostic markers is urgent.

Small nucleolar RNAs (snoRNAs) are small noncoding RNAs ranging from 60 to 300 nucleotides [5,6]. SnoRNAs are essential for numerous cellular processes and mainly execute their functions in the nucleolus [7]. SnoRNAs are classified according to their structures and function into C/D box and H/ACA box types. By binding different associated proteins and enzymes, C/D and H/ACA box snoRNAs become small nucleolar RNA-protein complexes (snoRNPs) and execute the site-specific methylation or pseudouridylation of rRNAs guided by snoRNAs [8,9]. It is well-known that snoRNA activity is responsible for the site-specific modification of rRNA, which directly contributes to translation fidelity and efficiency [10]. Although they have been considered as housekeeping RNAs for decades, the aberrant expression or activity of snoRNAs has been recently reported in human cancers [11,12,13]. Zhou et al. reported that increased C/D box snoRNAs and their 2′-O-methylation activity of rRNAs are required for leukemogenesis by impairing protein synthesis accuracy [14]. Loss of SNORA24-mediated pseudouridylation of rRNAs disrupts translation fidelity, which cooperates with RAS^G12V^ to promote liver cancer [15]. Decreased SNORD50-guided methylation on 28S rRNA impairs IRES-mediated translation in colon cancer [16]. SNORA42 is elevated in non-small cell lung cancer (NSCLC) patients, which is correlated with poor prognosis [17]. SNORD76 expression is lower in grade III/IV patients than in grade II patients and is considered a tumor suppressor in glioblastoma [17]. At the genomic level, SNORD50A (U50A) is a putative tumor suppressor since the somatic deletion of U50A is correlated with poor prognosis in breast cancer patients [18]. Although 20% of breast cancers have heterozygous deletions of the U50A locus in the genome, no homozygous deletion has been observed [19]. Through the protein interactome, U50A has been identified to have a noncanonical function in inactivating the KRAS protein [18]. However, the role of U50A RNA expression in breast cancer progression and its impact have not been fully-established, especially the resulting transcriptomic regulation and pathological functions.

## 2. Materials and Methods

### 2.1. Patient Specimens

We analyzed 114 samples of breast cancer tissues from individual patients. Specimens were obtained from surgical section during 2003–2013 by National Cheng Kung University Hospital (Tainan, Taiwan). Waivers of informed consent were approved by IRB A-ER-103-131 from NCKU Hospital. Frozen breast cancer tissues were used according to approved IRB (A-ER-103-131). Tumor stage was classified according to the American Cancer Society.

### 2.2. Total RNA Extraction

Total RNA was extracted using TRIzol (Invitrogen, Waltham, MA, USA) according to the manufacturer’s protocol. Briefly, chloroform was added, and homogenized samples were centrifuged at 14,000× *g* for 30 min. The clear upper aqueous layer was isolated and precipitated with isopropanol at −80 °C for 1–2 h. Samples were centrifuged at 14,000× *g* for 30 min, and the supernatant was discarded. The resulting pellets were washed with 75% ethanol and preserved in nuclease-free water at −80 °C. Total RNA concentrations were determined by spectrophotometer (NanoDrop Lite Microlitre Spectrophotometer, Thermo Fisher Scientific™, Waltham, MA, USA).

### 2.3. RNA-Sequencing Analysis

The purified RNA was used for the preparation of the sequencing library by TruSeq Stranded mRNA Library Prep Kit (Illumina, San Diego, CA, USA) following the manufacturer’s recommendations. Briefly, total RNAs from three independent biological replicates were collected and equally pooled for RNA-sequencing analysis. mRNA was purified from total RNA (1 μg) by oligo(dT)-coupled magnetic beads and fragmented into small pieces under elevated temperature. The first-strand cDNA was synthesized using reverse transcriptase and random primers. After the generation of double-strand cDNA and adenylation on 3′ ends of DNA fragments, the adaptors were ligated and purified with AMPure XP system (Beckman Coulter, Beverly, CA, USA). The quality of the libraries was assessed on the Agilent Bioanalyzer 2100 system (Agilent, Santa Clara, CA, USA) and a Real-Time PCR system. The qualified libraries were then sequenced on an Illumina NovaSeq 6000 platform with 150 bp paired-end reads generated by Genomics, BioSci & Tech Co., New Taipei City, Taiwan.

### 2.4. Reverse Transcription

Reverse transcription was performed using the RevertAid RT Reverse Transcription Kit (Thermo Fisher Scientific) according to the manufacturer’s protocol. A reaction containing 200 ng template RNA, 1 μL random hexamer, and 2 μL 10 mM dNTP mix with nuclease-free water adjusted to 12 μL was incubated at 65 °C for 5 min. A mixture of 4 μL 5X reaction buffer, 1 μL RNase inhibitor, and 1 μL RevertAid RT was added, the volume was brought 20 μL to with nuclease-free water, and the reactions were incubated at 25 °C for 5 min, followed by 42 °C for 60 min. Finally, the reactions were terminated at 70 °C for 5 min. cDNAs were preserved at −20 °C.

### 2.5. SnoRNA Detection and Absolute/Relative Real-Time PCR

SnoRNA detection has been described previously [18]. Briefly, the total RNA was extracted, and reverse transcription was performed. Specific primers used for detecting U50A have been described previously [18]: forward primer: 5′-TATCTGTGATGATCTTATCCCGAACCTGAAC-3′ and reverse primer: 5′-ATCTCAGAAGCCAGATCCGTAA-3′, U50B: forward primer: 5′-GAAACCTATCCCGAAGCTGA-3′ and reverse primer: 5′-TCAGAAGCCGAATCCGTACT-3′ and SNHG5: forward primer: 5′-AAAACGCCTTGGAGTGTGAC-3′ and reverse primer: 5′-TGAAGACAGCGCCATTGTTC-3′.

For absolute quantification, a standard curve was established using linearized U50A/U50B/SNHG5 plasmids. Ten-fold serially-diluted linearized plasmid dilutions were prepared, detected by specific primers, and the initial concentration was adjusted to 10^8^ copy numbers to achieve the final concentration within 40 cycles. For relative quantification, specific primers were used to detect specific targets and compared with the control group. Real-time PCR was performed in 48 well optical plates with three repetitions. Total volume consisted of 2.5 μL Fast SYBR™ Green PCR Master Mix (Thermo Fisher Scientific), 0.5 μL forward primer, 0.5 μL reverse primer, 0.5 μL nuclease-free water, and 1 μL template. The real-time PCR program was carried out according to the manufacturer’s protocol, which included 95 °C for 20 s to activate the polymerase, 40 cycles of 95 °C for 3 s, and 60 °C for 30 s.

### 2.6. Plasmid and Transfection

U50A (NR_002743.2), U50B (NR_003044.3) and SNHG5 (NR_003038.2) were synthesized and cloned into pLUX vector. The plasmids were transfected into MCF-7 cells using the HyFectTM DNA transfection reagent according to the manufacturer’s protocol (Leadgene Biomedical, Tainan, Taiwan). Briefly, 6 μg of plasmid was mixed with the cells and incubated for 20 min at room temperature. The cells were then added to dishes with fresh medium and incubated for 48 h.

### 2.7. SnoRNA Inhibition

SnoRNA detection has been described previously [18]. Briefly, customized oligonucleotides with specific modification based on Siprashvili et al. [18] were synthesized, and 10 μM anti-sense oligonucleotides was transfected using Invitrogen Lipofectamine™ 2000 according to the manufacturer’s protocol for 24 h.

### 2.8. Lentiviral Knockdown

Lentiviral system was used to perform target mRNAs knockdown from RNAi core (Academia Sinica, Taipei, Taiwan). Three plasmids, namely packaging plasmid (pCMVΔR8.91), envelope plasmid (pMD.G), and shRNA plasmid (pLKO.1 shRNA), were transfected into HEK293T cells with a proportion of 10:10:1. Supernatant containing viral particles was collected and filtered with 0.22 μM filter after 24 h. Cells were infected with virus medium and polybrene for 24 h and selected with puromycin (1.5 μg/mL) for 48 h.

### 2.9. Western Blot

Cells were harvested by RIPA buffer and fractionated by SDS-PAGE. Protein were transferred onto PVDF membranes according to the manufacturer’s protocols (Bio-Rad, Hercules, CA, USA). PVDF membrane was purchased from GE Healthcare (Uppsala, Sweden). After blocking with 5% nonfat milk in TBST for 60 min, membranes were washed and incubated with primary antibodies(GeneTex, Irvine, CA, USA), SMC5 (GTX115669), ATRX (GTX101310), CENPF (GTX100212), CENPE (sc-376685), Phospho ser28 histone H3 (GTX128953), Phospho ser10 histone H3 (GTX128116), and total histone H3 (GTX122148) at 4 °C overnight. Membranes were washed three times for 10 min and incubated with secondary antibodies for 60 min. Protein expressions were visualized by the ECL system according to the manufacturer’s protocols. ECL (Enhanced Chemiluminescent) was purchased from PerkinElmer (Waltham, MA, USA). For the original Western blots, see Figure S9.

### 2.10. Immunofluorescence

Cells were washed 3 times with cold PBS, fixed in 4% formaldehyde for 30 min, and then permeated with 0.5% Triton X-100 for 10 min. After being washed, cells were stained with DAPI for 10 min and kept in PBS. The images of nuclei were captured by fluorescence microscopy at indicated time points. Quantification of prometaphase, metaphase, and anaphase cells were calculated by ImageJ cell counter software (version 1.53m).

### 2.11. In Situ Hybridization (ISH) Detection

Breast cancer tissue arrays (Pantomics, Fairfield, CA, USA) were used for in situ hybridization (ISH) staining. Tissue sections were stained with a Dig-labeled U50A probe (sequence 5′-AGT TCA GGT TCG GGA TAA GAT CAT CAC AGA-3′) synthesized by BioTnA. U50A signals were detected by a Biospot ISH detection kit (TASH01D, BioTnA, Kaohsiung, Taiwan). The results were presented in DAB chromogen and observed using a microscope. All glass slides were digitized with an Motic Easyscan Digital Slide Scanner (Motic Hong Kong Limited, Hong Kong, China) at 40× (0.26 μM/pixel) with high precision (high precision autofocus). Motic Easyscan whole-slide images were viewed with DSAssistant and EasyScanner software (version 1.13). All tissue sections were analyzed and scored by a pathologist.

### 2.12. Statistical Analysis

We used a log-rank test in Kaplan–Meier curves for overall and relapse-free survival. The event used for overall survival is patient death, and the event of relapse-free survival included patient death and recurrence of tumor. Median was used as the cutoff to separate high and low group. The *t*-test was used to compare two group experiments. For multigroup analysis, one-way ANOVA with Tukey’s multiple comparisons test was used. Fisher’s exact test was used to analyze low and high U50A ISH score.

### 2.13. Colony Formation

A total of 2000–4000 breast cancer cells was seeded into 6-well plates. After 7–14 days, colonies were formed and fixed with 3.7% paraformaldehyde at room temperature for 0 min. The fixed colonies were washed and stained by 0.05% Coomassie blue for 15 min at room temperature. The colonies in every well were counted using ImageJ software (version 1.53m).

## 3. Results

### 3.1. U50A Expression Level Is Associated with Better Prognosis in Breast Cancer Patients

**Figure 1:**
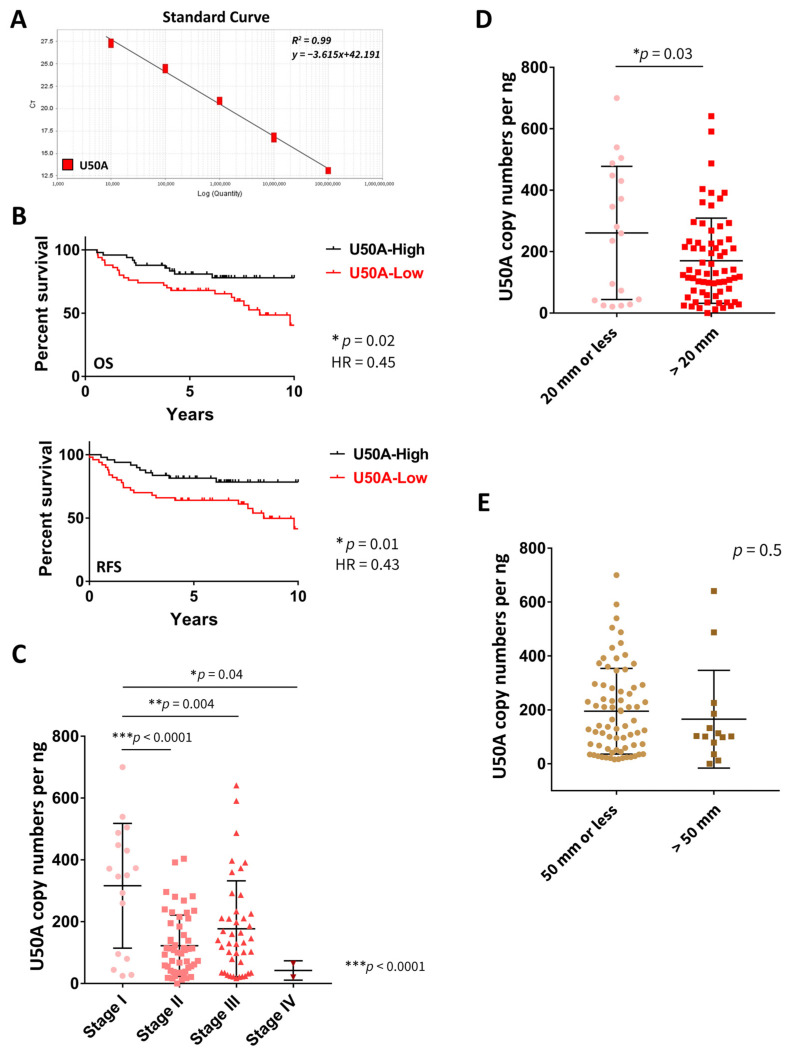
U50A expression is associated with better prognosis in breast cancer patients. (**A**) Absolute quantification of copy number of U50A, snoRNA. The standard curve of U50A was obtained using plasmid template. Ten-fold serial dilutions of plasmid templates were used. (**B**) Kaplan-Meier curves for overall survival (OS, top) and relapse-free survival (RFS, bottom) of breast cancer patients with U50A-high and U50A-low. Median was used as the cutoff to separate U50A-high and U50A-low groups. Correlation between the snoRNA copy numbers of U50A and the tumor stage (**C**), and tumor size divided by 20 mm (**D**) or 50 mm (**E**) of breast cancer patients. * *p* < 0.05, ** *p* < 0.01, *** *p* < 0.001.

U50A is considered to be a putative tumor suppressor because the somatic deletion of its gene locus is correlated with poor prognosis in breast cancer patients [18]. However, the role of U50A RNA expression in breast cancer patients is unclear. In this study, we first used absolute quantitative real-time PCR using a ten-fold serial dilution of linearized plasmid templates to quantify the U50A snoRNA copy number. The final range of the standard curve was established from 10^4^ to 10^8^ U50A copy numbers. The R^2^ value was 0.99 for U50A (Figure 1A). In our study, tissues from breast cancer patients were collected with the clinical and pathological characteristics of the patients listed in Table 1. Consistent with previous studies, significant reduction in U50A RNA levels was observed in tumors compared to paired normal tissues (Figure S1), which suggested the potential diagnostic application of tumor suppressor U50A in the future. Next, we determined the U50A snoRNA copy number to analyze its correlation with survival of breast cancer patients. The median was used as the cutoff for separating patients into high and low U50A expression groups. We found that higher U50A expression was significantly correlated with better overall survival (OS) in breast cancer patients (*p* = 0.02, HR = 0.45; Figure 1B, top and Table 2). Longer relapse-free survival (RFS) was also observed in breast cancer patients with elevated U50A expression (*p* = 0.01, HR = 0.43; Figure 1B, bottom and Table 2). These data indicated that the U50A copy number is significantly predictive of breast cancer patient survival. U50B is a snoRNA in the adjacent gene locus of U50A, and snoRNA host gene 5 (SNHG5) is the host gene of both U50A and U50B. Therefore, we also investigated the correlation between copy numbers of U50B or SNHG5 and breast cancer patient survival. The standard curve was confirmed from 10^3^ to 10^7^ U50B copy numbers and from 10^2^ to 10^8^ SNHG5 copy numbers. Both of the R^2^ values were 0.99 (Figure S2). No significant difference between high U50B and low U50B expression of overall and relapse-free survival in breast cancer patients was observed (Figure S2A and Table 2). Expression of SNHG5 showed no correlation with survival in breast cancer patients (Figure S2B and Table 2). Moreover, we included a breakdown of the clinical molecular subtypes including luminal A/B, HER2-enriched, and triple negative breast cancer (TNBC) (Figure S3 and Table S1). Although the *p* values of HER2 and TNBC subgroups were limited due to patient number, U50A expression was associated with better prognosis among all the subtypes, which was similar to the trends observed in all breast cancer patients.

We next investigated the relationships between U50A expression and pathological parameters. In breast cancer patient tissues, the U50A copy number was dramatically lower in stage 2 to stage 4 tumors compared to stage 1 tumors (Figure 1C and Table 3; *p* < 0.0001, *p* = 0.004, and *p* = 0.04, respectively). We also analyzed these tumors according to their tumor size either by 20 mm or 50 mm, which are the clinical parameters in defining T stages. U50A expression was significantly reduced in tumors >20 mm compared to tumors ≤20 mm, but no significant changes between tumors >50 mm and ≤50 mm were observed (Figure 1D,E). Relationships between U50B or SHNG5 and stage or tumor size were analyzed in Figure S4. Moreover, the breakdown of clinical molecular subtypes was also analyzed (Figures S5–S7), and similar results of low U50A in association with advanced tumor stages were found in both luminal A/B and TNBC subgroups (Figures S5A and S7A and Table S2), though there were only limited stage I TNBC patients. Interestingly, we unexpectedly observed that low U50B significantly correlated with advanced tumor stages, specifically in the TNBC subgroup (Figure S7B and Table S2), leaving the possibility that U50B may have clinical or pathological impacts in TNBC. SNHG5 also showed borderline significant decreases in tumor stages in luminal A/B subgroups (Figure S5C and Table S2). To determine whether U50A is an independent prognosis marker, multivariate analysis using U50A with other standard covariates, including expression of T, N, and stage status was performed. The results of multivariate cox regression analysis indicated that U50A expression is associated with reduced hazard ratio (OS, HR = 0.519; RFS, HR = 0.480), even though the *p* values were not less than 0.05 (OS, *p* = 0.098; RFS, *p* = 0.06) (Table S3). Taken together with the abovementioned results, low U50A expression was observed in advanced stages of breast cancer and correlated with tumor size, suggesting its potential role contributing to the regulation of tumorigenesis.

### 3.2. Mitosis-Related Transcriptomic Changes Regulated by U50A

**Figure 2:**
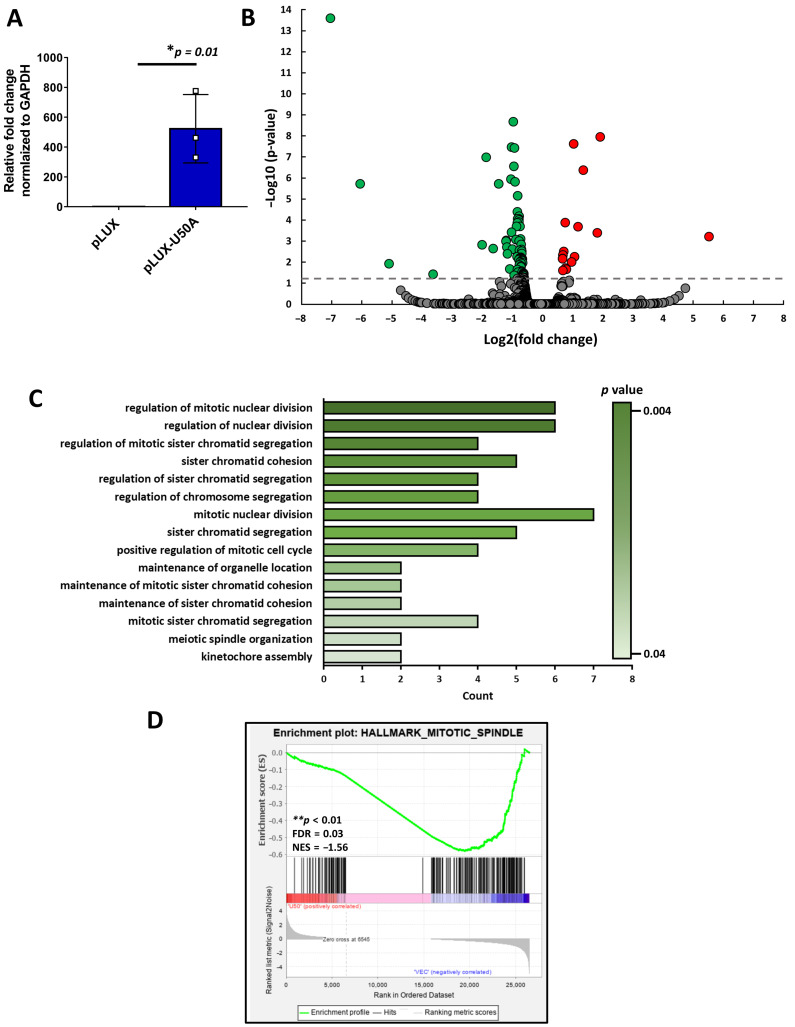
Transcriptomic changes regulated by U50A. (**A**) Fold change of U50A in samples used for RNA sequencing. * *p* < 0.05. (**B**) Volcano plot of RNA-sequencing results. Green and red dots represent significantly downregulated and upregulated genes, respectively. The gray line indicates the *p* value of 0.05. (**C**) Bar chart of significantly downregulated Gene Ontology terms in the biological process category. (**D**) Enrichment plot of the GSEA results in the hallmark gene set (NOM *p* < 0.01; FDR = 0.03; NES = −1.56) ** *p* < 0.01.

To explore the pathological function of U50A, we overexpressed U50A in MCF-7 cells and subjected them to RNA sequencing analysis. The relative expression of U50A RNA showing the successful overexpression was confirmed by quantitative real-time PCR (Figure 2A). We used RNA-sequencing data to analyze the transcriptomic changes in U50A-overexpressing cells. The volcano plot depicts the log2 (fold change) versus-log10 (*p*-value) for visualizing the significantly regulated genes. The gray line indicates statistically significant genes, and the green/red dots indicate decreased/increased genes (Figure 2B). We observed more significantly downregulated genes (green dots, 72/87; 82.76%) than upregulated genes (red dots, 15/87; 17.24%) (Figure 2B), suggesting the role of U50A in the inhibition of cellular pathways. Consistent with a previous study indicating the U50A-suppressed RAS/RAF/ERK pathway, downregulated multiple MAPK pathways were enriched in RNA sequencing result comparing U50A overexpression to control [18]. Furthermore, to investigate the functional consequences of U50A overexpression, we used Gene Ontology to analyze the downregulated genes. The significantly decreased categories listed in the bar chart arranged by *p*-value (Figure 2C) showed that most of the categories are related to nuclear division or the regulation of chromatid segregation, which indicates the ability of U50A to suppress the mitosis process (Figure 2C). Gene set enrichment analysis (GSEA) is another analytical approach developed by Subramaniana that evaluates RNA-sequencing data at the level of gene sets/pathways [20]. Therefore, we also subjected the transcriptomic profile for GSEA analysis, and a similar result was observed. The mitotic spindle category, which contains the genes important for mitotic spindle assembly in the hallmark gene set, was dramatically decreased (Figure 2D). During mitosis, there are numerous phases that carry out different processes to complete mitosis [21]. DNA duplication, chromatin condensation, and nuclear division are included in mitosis [22]. Mitotic spindles are also indispensable for cell division [22]. Hence, these results suggested that U50A downregulates genes in mitotic-related categories and may further negatively regulate cells during mitosis.

### 3.3. Identification of the Downstream Targets of U50A Involved in Mitosis

**Figure 3:**
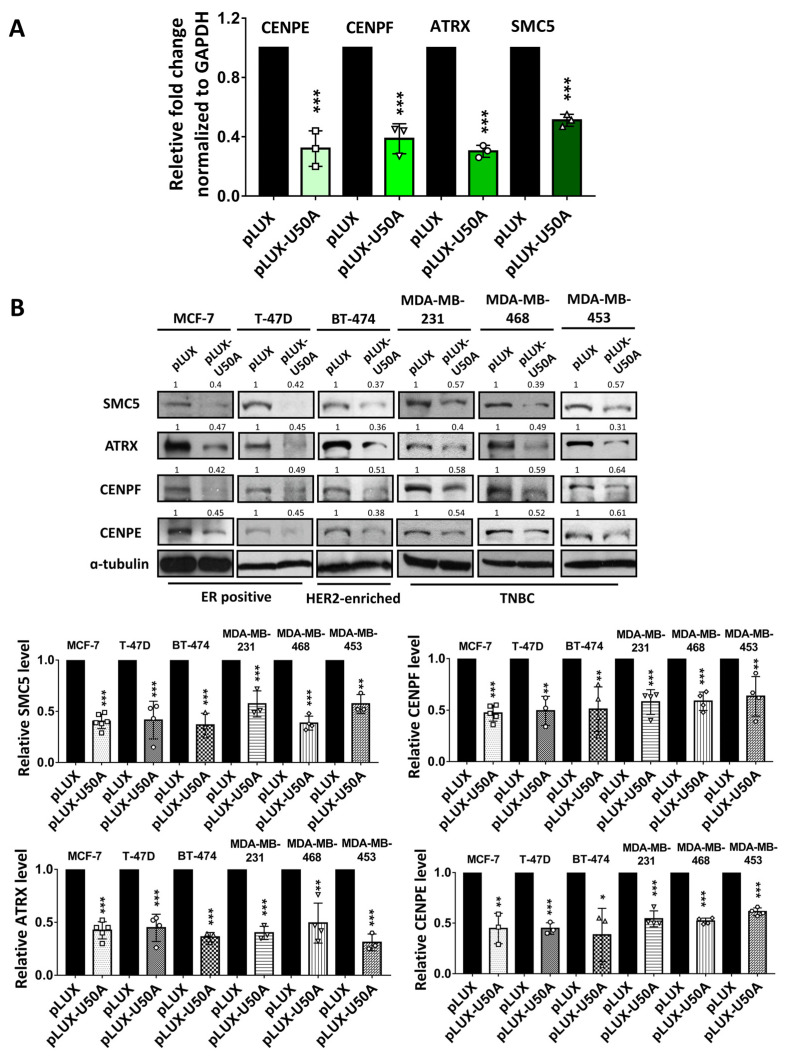

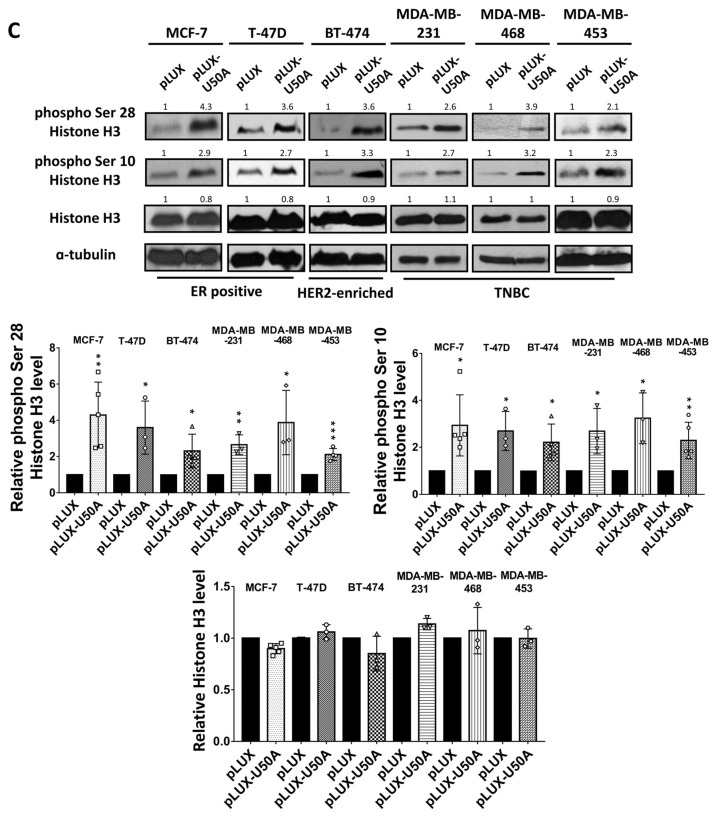

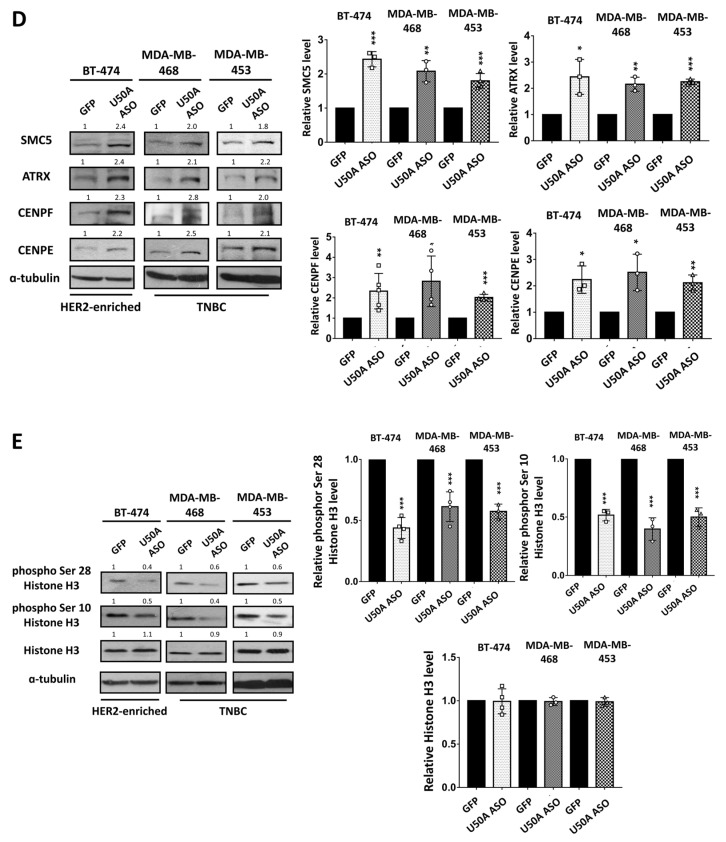
Mitosis-related genes regulated by U50A. (**A**) Validated fold change of CENPE/F, ATRX, and SMC5 in U50A-overexpressing MCF-7 cells. Data are represented as the mean ± SD (*n* ≥ 3). *** *p* < 0.001. Protein expression of the selected targets in U50A-overexpressing (**B**) and U50A-inhibiting (**D**) breast cancer cell lines. Mitotic markers in U50A-overexpressing (**C**) and U50A-inhibiting (**E**) breast cancer cell lines. Bar chart were three independently repeated data quantitated by Image J. Cells were harvested and subjected to real-time quantitative PCR (**A**) and Western blotting (**B**–**E**). * *p* < 0.05, ** *p* < 0.01, *** *p* < 0.001. The full Western Blots images can be found in Figure S9.

The transcriptomic and bioinformatics analysis showed that U50A downregulates a group of genes that mediate multiple pathways related to mitosis (Figure 2), while the mitotic spindle category from GSEA also showed similar results (Figure 2D). Four candidate genes including SMC5, ATRX, CENPE, and CENPF were further selected after overlapping these functional categories (Table 4). Of these candidate downstream genes, SMC5, or structural maintenance of chromosomes protein 5, is a member of the SMC complex that has been reported to regulate chromatin organization [23,24]. ATRX, α-thalassemia mental retardation X-linked protein, is a helicase with ATPase activity that belongs to chromatin remodeling protein [25]. CENPE and CENPF, centromere protein E and F, respectively, are two proteins involved in chromone segregation, kinetochore-microtubule conjugation, and spindle assembly during mitosis [26]. To validate the RNA-sequencing data, we analyzed three independent repeats of U50A overexpression samples to investigate the RNA and protein levels of these genes. The data showed that SMC5, ATRX, CENPE, and CENPF are decreased in U50A-overexpressing cells, which is consistent with the RNA-sequencing results (Figure 3A and Table 4). Next, we validated the protein expression of these genes in multiple breast cancer cell lines, including MCF-7 and T-47D (luminal A/B), BT-474 (HER2-enriched), MDA-MB-231, MDA-MB-468, and MDA-MB-453 (TNBC). The fold changes of U50A overexpression were confirmed, as shown in Figure S8A. The result showed that U50A consistently downregulates SMC5, ATRX, CENPE, and CENPF, indicating a widespread phenomenon that these genes are downstream targets of U50A in breast cancer (Figure 3B). These U50A-downregulated genes are known to be essential for cell–cell division, which implies the potential ability of U50A in suppressing cell mitosis. The phosphorylation of histone H3 occurs during mitosis and is conserved in eukaryotes [27,28,29]. Histone H3 Ser10/28 phosphorylation is a feature of condensed chromatin, which is maintained from the late G2 to M phase, and eventually dephosphorylated at the telophase [29]. Therefore, we investigated the phosphorylation of these common mitotic markers, histone H3 Ser10/28, in U50A-overexpressing cells. Interestingly, we observed elevated phospho Ser10 and 28 histone H3, which indicates increased mitotic cell numbers in U50A-overexpressing cells (Figure 3C). The increased expression of mitotic markers suggests two possibilities: the induced entry of the G2/M phase or the prolonged mitotic phase. The upregulated cell cycle triggers cells to enter the G2/M phase, resulting in elevated mitotic cell numbers; on the other hand, prolonged mitosis arresting cells in the M phase may also exhibit the same phenomenon. Considering its tumor suppressive activity and the target genes we identified, U50A was proposed to downregulate mitosis-related genes that cause cell cycle arrest in the prolonged M phase. To confirm these phenomena, we used U50A antisense oligonucleotide to inhibit U50A expression [18]. We first analyzed relative expression of U50A in a panel of breast cancer cell lines, and result showed that U50A expression level are relatively high in BT474, MDA-MB-468, MDA-MB-453, MDA-MB-231, and Hs578T cells compared to MCF-7 cells (Figure S8B). Since MDA-MB-231 and Hs578T were reported as U50A mutant cell lines [19], we chose BT-474, MDA-MB-468, and MDA-MB-453 as our model cell lines for U50A inhibitor treatment (Figure S8C). SMC5, ATRX, and CENPE/F were upregulated in U50A-inhibited cells, and phospho Ser10/28 histone H3 were downregulated, which showed consistent results upon U50A overexpression (Figure 3D,E). The results once again proved the inhibitory effect of U50A in mitosis-related genes, SMC5, ATRX, and CENPE/F, and the prolonged M phase in the cell cycle.

### 3.4. U50A Prolongs Mitosis and Suppresses Colony-Forming Ability in Breast Cancer Cells

**Figure 4:**
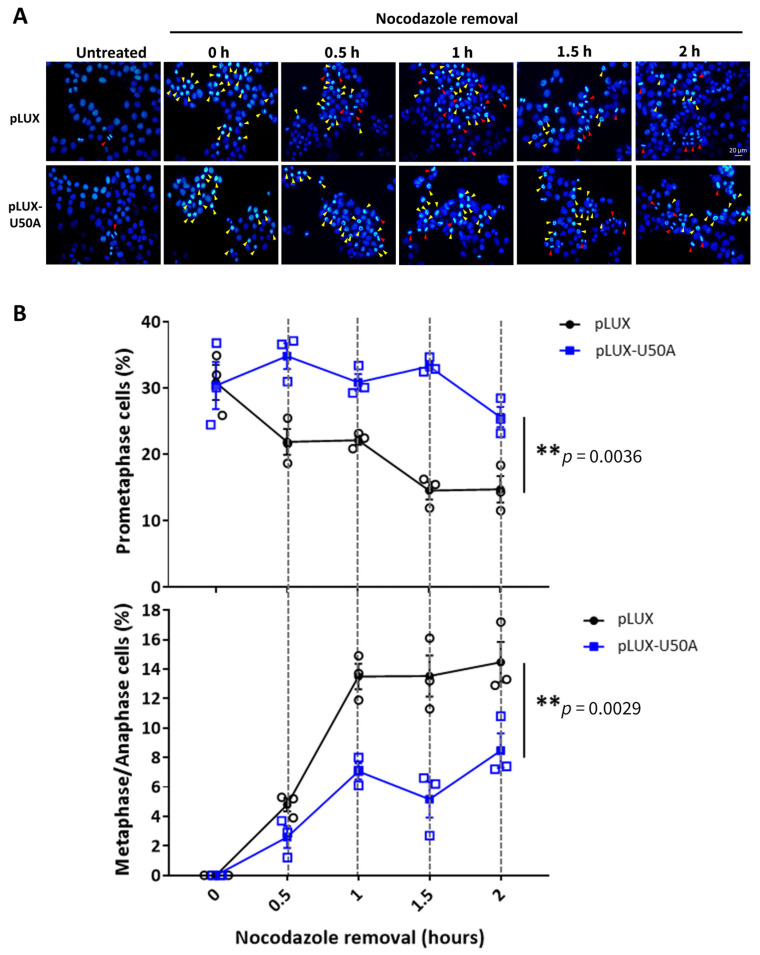

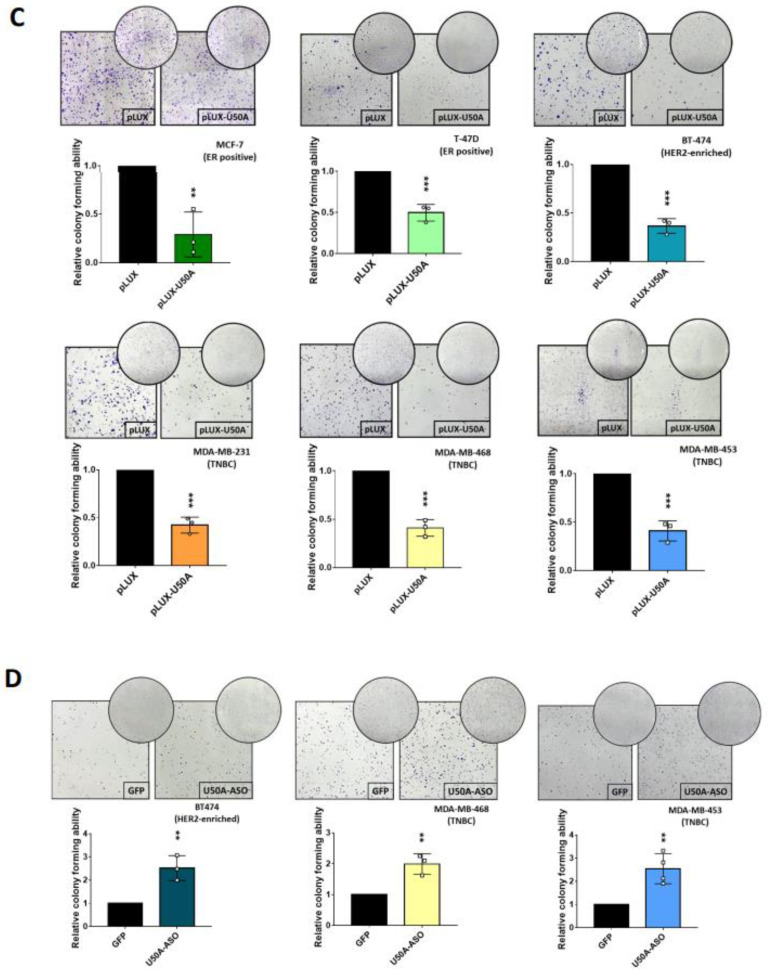

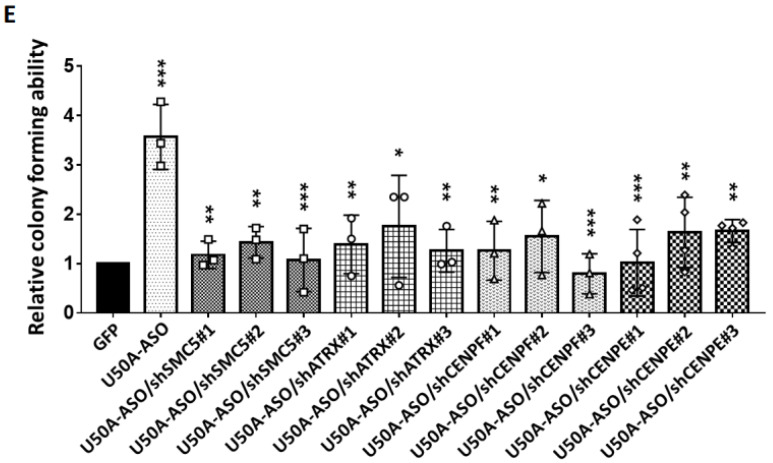
Effects of U50A on mitosis and colony-forming ability of breast cancer cells. Nocodazole (100 nM) was used to treat U50A-overexpressing MCF-7 cells for 14 h and was removed at the indicated time points. Cells were collected and subjected to immunofluorescence. (**A**) Representative images of immunofluorescence in U50A-overexpressing and control MCF-7 cells at the indicated time points. Yellow arrowheads indicate prometaphase cells, and red arrowheads indicate metaphase/anaphase cells. (**B**) Quantitated results of prometaphase, metaphase, and anaphase cells from immunofluorescence images. Colony forming ability of breast cancer cells expressing U50A (**C**) and inhibiting U50A (**D**) were assayed. (**E**) Specific shRNAs were used to inhibit CENPE/F, ATRX, and SMC5 in U50A-inhibiting BT-474 cells and colony forming capacity were assayed. Data are represented as the mean ± SD (*n* ≥ 3). * *p* < 0.05, ** *p* < 0.01, *** *p* < 0.001.

Mitosis is the process by which a single cell divides into two cells [21,22]. Prophase, prometaphase, metaphase, anaphase, and telophase progress sequentially during mitosis [22]. Chromatin condensation is the crucial first step in prophase; next, in prometaphase, nuclear envelope fragmentation leads to the spread of condensed chromatin covering the cells. Next, chromatin lies in the middle of cells ready for division in metaphase and is pulled over to opposite sides of the cell in anaphase. Finally, in telophase, a single cell divides into two cells, thereby finishing the mitosis process [22]. Since we hypothesized that U50A extends the mitosis time, we applied nocodazole which prevents microtubule polymerization and is widely used in cell cycle analysis to U50A-overexpressing cells [30]. Therefore, we used nocodazole to synchronize mitotic cells in prometaphase and removed it to investigate the mitosis duration time. Due to the unique type of chromatin in different phases, DAPI staining alone is sufficient to differentiate each phase in mitosis [31,32]. Cells in the untreated group showed limited mitotic cells under the microscope (Figure 4A, red arrowhead in left panel). After treatment with nocodazole, approximately 50% of cells were synchronized in prometaphase, which showed that the high-intensity DAPI signal covered the cells (Figure 4A, yellow arrowheads in the 0 h group). Then, we removed nocodazole and harvested cells at the indicated time points to observe the duration of mitosis. A single stick-shaped DAPI signal represented the metaphase cells; the anaphase cells displayed two stick-shaped DAPI signals lined up with each other. We observed that the metaphase/anaphase cells increased dramatically after 1 h of nocodazole removal in the pLUX group (Figure 4A, red arrowheads on top), with decreased prometaphase cells (Figure 4A, yellow arrowheads on top). However, only a small portion of the cells entered metaphase/anaphase (Figure 4A, red arrowheads at bottom), and most of the cells were arrested in prometaphase in the U50A overexpression group (Figure 4A, yellow arrowheads at bottom). Cells exiting from prometaphase were slower in U50A-overexpressing cells than in control cells, indicating that U50A prolongs the prometaphase of mitotic cells (Figure 4B, top panel). Simultaneously, the percentage of metaphase/anaphase cells was decreased in U50A-overexpressing cells, which also supported that U50A prevents cell exit from prometaphase and leads to reduced entry of metaphase/anaphase (Figure 4B, bottom panel). To obtain the molecular effect of the phenomenon that U50A expression is reduced in larger tumor size (Figure 1D), we next investigated the effect of U50A in colony forming ability in breast cancer cell lines. In U50A-overexpressing cells, downregulated colony forming ability was observed in multiple breast cancer cell lines, and consistent results were observed in U50A-inhibited cells (Figure 4C,D). Prolonged M phase decreases cell proliferation rate, which results in attenuated clonogenic activity [33]. To study the functional involvement of SMC5, ATRX, and CENPE/F in U50A-mediated phenotype under endogenous circumstance, we repressed CENPE, CENPF, ATRX, and SMC5 expression, which were upregulated by U50A inhibition. U50A inhibition was followed by knocking down individual downstream genes for colony formation assays. SMC5, ATRX, and CENPE/F were individually knocked down using specific short-hairpin RNAs (shRNAs) in BT-474 cells. The data showed that U50A inhibition-enhanced colony-forming ability was further mitigated by knockdown of SMC5, ATRX, or CENPE/F (Figure 4E). These results validated the functional role of these downstream genes, and U50A-mediated suppressed mitosis may regulate the initiation of breast cancer tumorigenesis in vitro.

### 3.5. Correlation of U50A Expression with Mitotic Cells in Human Breast Cancer Tissues

**Figure 5:**
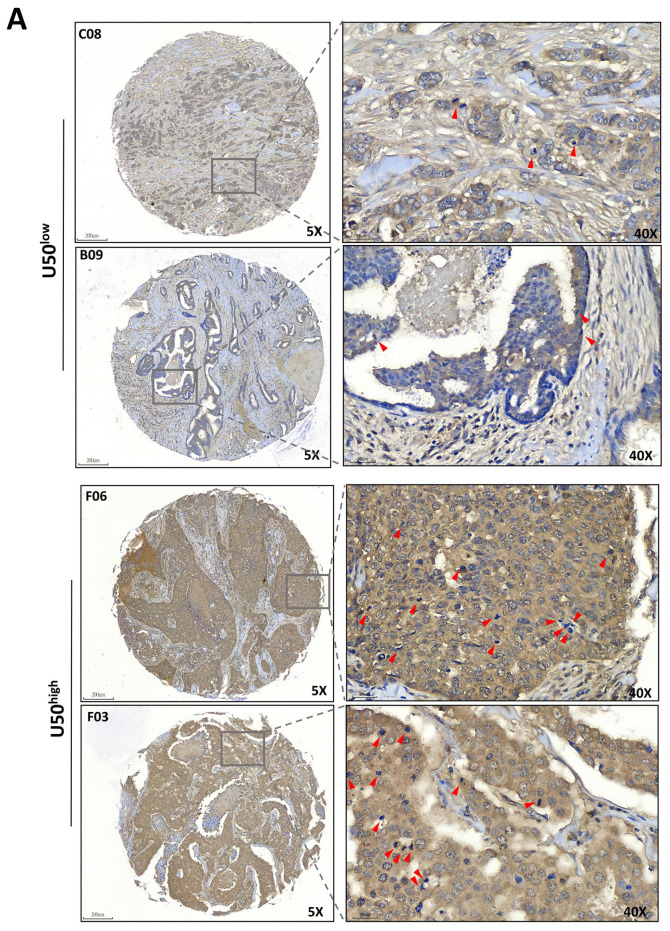

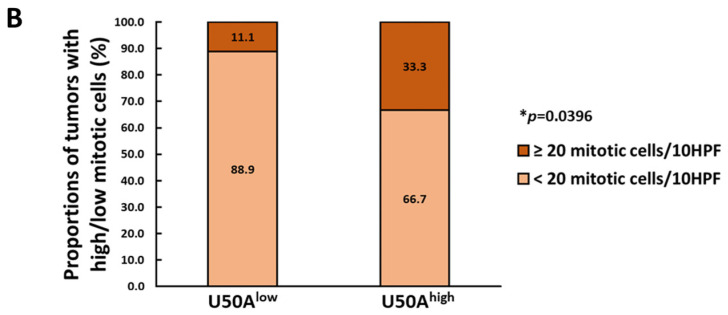
Correlation between U50A and mitotic cells in breast cancer patients. A breast cancer patient tissue array (BRC1021) was applied for in situ hybridization (ISH) of U50A. (**A**) Representative images of U50A in situ hybridization. The top and bottom panels show the U50A-low/U50A-high breast cancer patients, respectively, and enlarged images are on the right. Red arrowheads indicate mitotic cells. Scale bar = 200 μm (5×); 30 μm (40×). (**B**) Quantitated result of mitotic cells in U50A-low/high samples from ISH images. HPF, high-power field. Fisher’s exact test was used. * *p* < 0.05.

**Figure 6:**
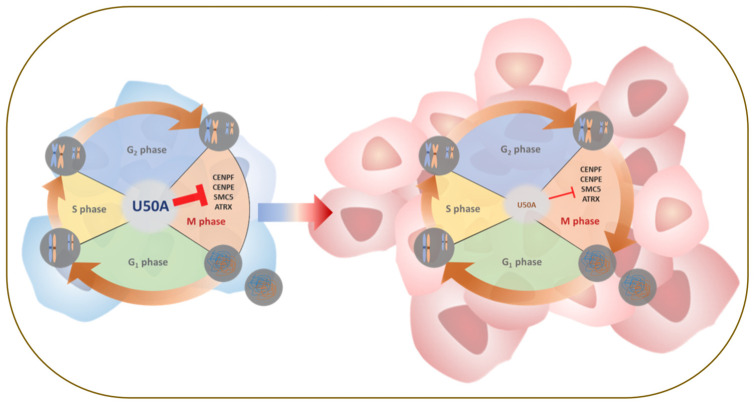
Schematic representation of U50A-prolonged mitosis.

To investigate these phenomena in clinical samples, we performed in situ hybridization (ISH) of U50A in a breast cancer patient tissue array (BRC1021). Tissues were divided into low U50A and high U50A groups according to the ISH score, and mitotic cells were counted using hematoxylin stain. In the low U50A group, fewer mitotic cells were observed (Figure 5A, red arrowheads on the top), whereas accumulated mitotic cells were observed in the high U50A group (Figure 5A, red arrowheads on the bottom). Quantitative analysis of the U50A ISH score and proportions of tumors with high/low mitotic cells showed similar results: the higher U50A ISH scores were associated with increased percentage (33.3%) of ≥20 mitotic cells compared with that (11.1%) of U50A^low^ tumors (Figure 5B, *p* = 0.0396), and vice versa. In conclusion, we used absolute real-time PCR to demonstrate U50A as a better prognostic marker in breast cancer (Figure 1) and found that U50A downregulates the mitosis-related genes SMC5, ATRX, CENPE, and CENPF, which arrest cells in prometaphase, leading to prolonged M phase and downregulated clonogenic activity (Figure 2, Figure 3 and Figure 4). In the breast cancer tissues, the number of mitotic cells was also elevated in the high U50A group (Figure 5). From in vitro to clinical studies, we demonstrated the tumor-suppressive function of U50A in inhibiting mitosis and that higher U50A expression is correlated with better breast cancer survival (Figure 6).

## 4. Discussion

Breast cancer is the most common cancer in women. Although it has a high incidence rate, the early detection of breast cancer can prolong the five-year survival rates. Traditionally, diagnostic methods such as mammography and breast ultrasound have been used for the early detection of breast cancer. Furthermore, the current prognostic markers used in daily practice are still the classical pathological parameters: TNM staging and ER, PR, and HER-2 receptor status. However, the shortcomings of current clinical practice include that detection is not early enough, and no specific molecular markers are available for the prediction of breast cancer prognosis. Therefore, our study established U50A as a biomarker for breast cancer that significantly discriminates overall survival and relapse-free survival in breast cancer. Moreover, during breast cancer progression, U50A expression decreases. These clinical data also reflect the molecular function of U50A in inhibiting tumorigenesis.

SnoRNAs are nucleolar-localized noncoding RNAs that are highly conserved among species. Previously identified as a class of housekeeping RNAs, snoRNAs execute their functions to maintain numerous cellular processes. Some snoRNAs have noncanonical functions and may play roles in regulating cancer progression. In recent years, the aberrant expression of snoRNAs in cancer has been reported. SNORA42, which is elevated in non-small cell lung cancer (NSCLC) patients, is considered a poor prognostic marker [34]. On the other hand, SNORD76 inhibits cell proliferation and is downregulated in glioblastoma [17]. Emerging research implies that extensively dysregulated noncoding RNAs may be an index for abnormal cell appearance. U50A locus deletion correlates with poor prognosis in breast cancer, which suggests that U50A is a tumor suppressor [18]. U50A snoRNA expression has been found to inactivate KRAS, which confirms its biological function in vitro [18]. However, the clinical relevance of U50A expression is unclear. Our results show that the U50A copy number is associated with better prognosis that successfully differentiates breast cancer patient survival, indicating that there is a hidden factor behind the U50A copy number. Therefore, we explored the function of U50A by RNA sequencing and found that U50A downregulates four mitotic-related genes, SMC5, ATRX, and CENPE/F. We observed that cells present a prolonged prometaphase resulting in delayed mitosis after U50A overexpression. In the breast cancer tissue array, high U50A expression also showed a high number of mitotic cells.

Current research about the pathological functions and regulation of mitosis-related genes CENPE/F, SMC5, and ATRX remain elusive. First, CENPE is overexpressed in lung adenocarcinoma and promotes lung cancer cells proliferation [35], which is regulated by FOXM1. Interestingly, we also observed that FOXM1 is the potential transcription factor binding to promoter regions of CENPE and CENPF (ChIP-Atlas), hypothesizing a mechanism that FOXM1 is the master regulator that simultaneously maintains the expression of these downstream genes under the control of U50A. Another possible regulatory mechanism mediated by U50A was proposed based on the previous findings that U50A mitigates the interaction between KRAS and CAAX farnesyltransferase (FTase). Since farnesylation is a known post-translational modification of CENPE/F proteins [36,37], it is also possible for U50A to affect the recognition of CENPE/F by FTase and further regulate their functions. The potential oncogenic role of ATRX in tumorigenesis is reported as a marker for defining the molecular subtype of glioma [25]. SMC5/6 complexes are essential for sumoylation of telomere-binding proteins to maintain telomere length in cancer cells utilizing alternative lengthening of telomeres (ALT) [24]. However, the upstream regulator of ATRX and SMC5 remains unclear, while our study provided the emerging evidence that U50A suppresses these genes to hinder cancer cell mitosis.

## 5. Conclusions

In summary, U50A expression can be used to differentiate breast cancer patient survival. Higher expression of U50A is correlated with better overall survival and relapse-free survival in breast cancer. We also revealed that U50A suppresses cell division by interfering with mitosis. Along with tumor progression, we demonstrated the tumor suppression activity of U50A in inhibiting tumorigenesis, which is consistent with the clinical patient data. In this study, we reveal new functions of U50A from molecular and clinical insights.

## Figures and Tables

**Table 1:** Clinicopathological characteristics in the study.

Characteristics	No. of Patients (%)
Median age (range, years)	50 (31–84)
Age ≤ 50	52 (46%)
Age > 50	62 (54%)
Pathologic characteristic	-
Invasive ductal carcinoma	97 (85%)
Invasive lobular carcinoma	1 (1%)
Others	16 (14%)
Others: Mucinous carcinoma, papillary carcinoma, and invasive carcinoma	
Breast cancer subtype	-
Luminal A/B	68 (60%)
HER2-enriched	28 (24.6%)
Triple-negative	18 (15.4%)

Frozen tissues were obtained from surgical resection during 2003–2013.

**Table 2:** Survival analysis of U50A, U50B, and SNHG5 in breast cancer patients.

Variable	All Breast Cancer Patients in This Study			
	Overall Survival (OS)		Relapse-Free Survival (RFS)	
	*p*	HR	*p*	HR
U50A	0.02 *	0.45 ^#^	0.01 *	0.43 ^#^
U50B	0.32	0.7	0.36	0.72
SNHG5	0.2	0.64	0.22	0.65

Survival analysis was analyzed by Log-rank test. ^#^ Hazard ratio (HR) < 0.5. * *p* < 0.05. Original data from Figure 1B and Figure S1.

**Table 3:** Correlation between copy numbers of U50A, U50B, or SNHG5 with pathological stages of breast cancer patients.

All	Stage (Mean ± S.D.)				
Marker	I	II	III	IV	*p*
U50A	316.3 ± 201.7	122.3 ± 98.98	177.1 ± 155.3	42.2 ± 31.4	*** <0.0001
U50B	10.48 ± 17.28	6.38 ± 10.86	5.005 ± 7.4	1.705 ± 2.39	0.62
SNHG5	36.04 ± 44.1	19.07 ± 38.69	60.72 ± 117.1	0.49 ± 0.67	0.07

S.D.: Standard error of the mean, *** *p* < 0.001, stage according to the recommendations of the American Cancer Society, using one-way ANOVA, Tukey’s multiple comparisons test, original data from Figure 1C and Figure S3A.

**Table 4:** Fold change of mitosis-related genes in RNA-sequencing analysis.

Selected Targets	Fold Change from RNA-Sequencing (pLUX-U50A/pLUX)
CENPE	0.49
CENPF	0.58
SMC5	0.63
ATRX	0.65

## Data Availability

All images and raw data available on request. All other data are available from the corresponding authors.

## Supplementary Materials

The following are available online at https://www.mdpi.com/article/10.3390/cancers13246304/s1, Figure S1: U50A expression in paired tumor and normal counterpart (Normal) tissues, Figure S2: Survival analysis of U50B and SNHG5 in breast cancer patients, Figure S3: Survival analysis of U50A, U50B, and SNHG5 in individual breast cancer subtypes, Figure S4: Correlation between clinical characteristics and U50B and SNHG5 in breast cancer patients, Figure S5: Correlation between clinical characteristic and U50A, U50B, and SNHG5 in luminal A/B breast cancer patients, Figure S6: Correlation between clinical characteristic and U50A, U50B, and SNHG5 in HER2-enriched breast cancer patients, Figure S7: Correlation between clinical characteristic and U50A, U50B, and SNHG5 in TNBC patients, Figure S8: U50A expression in modulated groups, Figure S9: Uncropped WB for Figure 3B–E, Table S1: Survival analysis of U50A, U50B, and SNHG5 in breast cancer subtypes (number of patients), Table S2: Correlation between copy numbers of U50A, U50B, or SNHG5 with pathological stages in breast cancer subtypes, Table S3. Multivariate analysis of breast cancer prognostic markers.
