# Supplementary material for: Expression of SnoRNA U50A Is Associated with Better Prognosis and Prolonged Mitosis in Breast Cancer
Source: Cancers (Basel). 2021 Dec 15;13(24):6304. doi: 10.3390/cancers13246304 (PMC8699759; doi:10.3390/cancers13246304)
Supplement: Supplementary file 1 [file cancers-13-06304-s001.zip › cancers-1449785-supplementary.pdf]

cancers

MDPI

Supplementary Materials: Expression of SnoRNA U50A Is Associated with Better Prognosis and Prolonged Mitosis in Breast Cancer

Jie-Ning Li, Ming-Yang Wang, Yi-Ting Chen, Yao-Lung Kuo and Pai-Sheng Chen

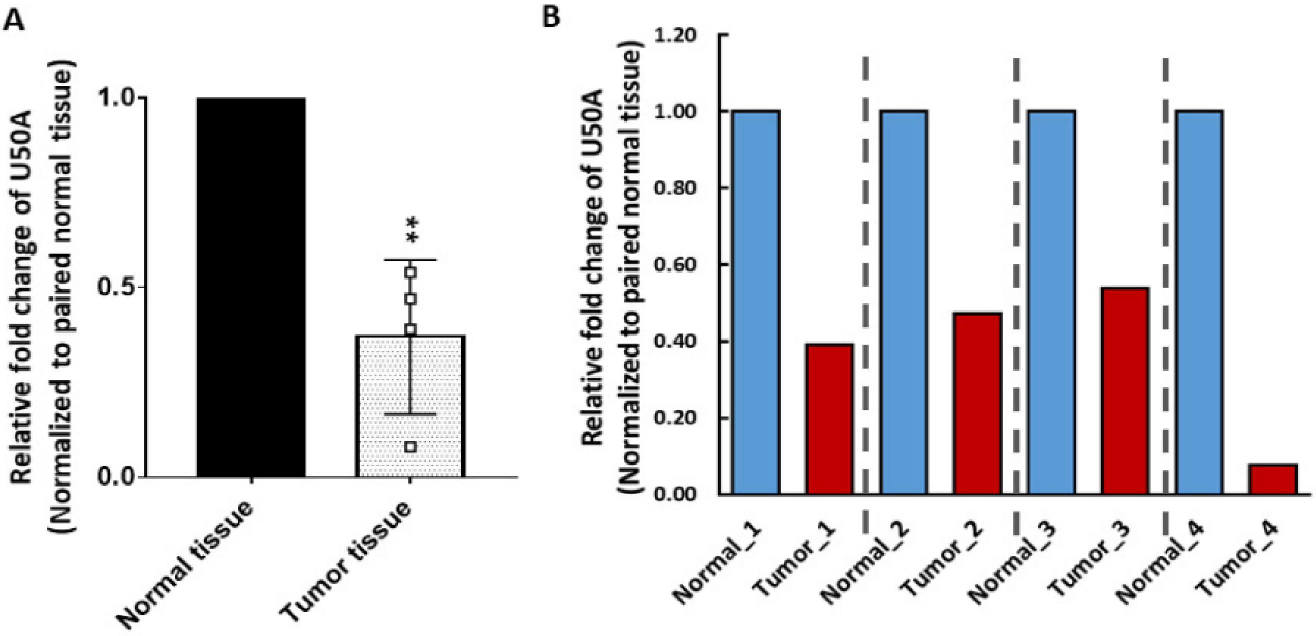

Figure S1. U50A expression in paired tumor and normal counterpart (Normal) tissues. U50A expression in four paired normal and tumor tissues from the same patients was analyzed by qPCR. (**A**) Relative fold changes were shown and was statistically analyzed using paired t-test (** $p < 0.01$). U50A expression in individual paired normal and tumor tissues were shown in (**B**).

A

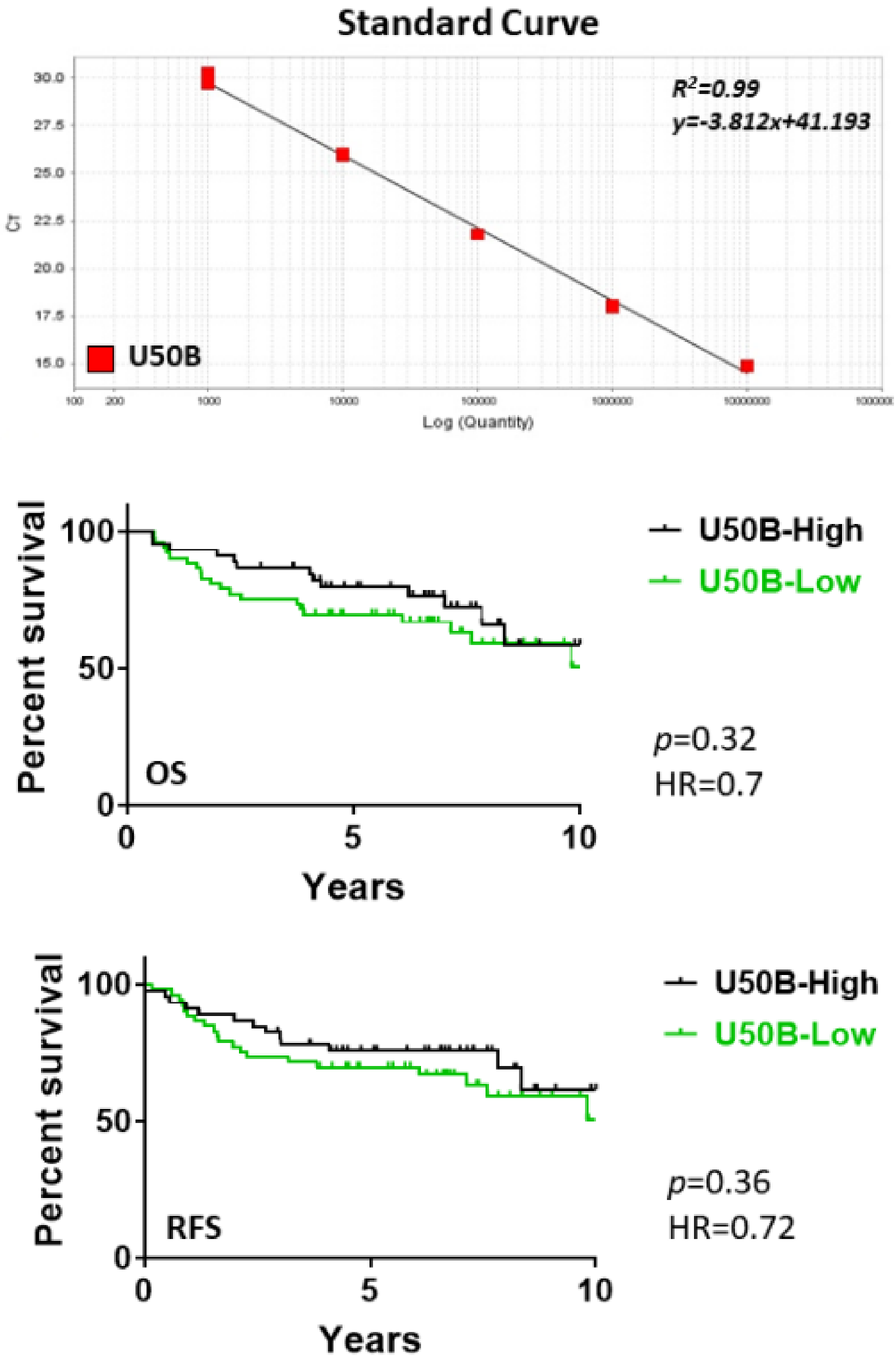
Standard Curve
R^2=0.99
y=-3.812x+41.193
Ct
30.0
27.5
25.0
22.5
20.0
17.5
15.0
U50B
100
200
1000
10000
100000
1000000
10000000
Log (Quantity)
Percent survival
100
50
0
OS
0
5
10
Years
U50B-High
U50B-Low
p=0.32
HR=0.7
Percent survival
100
50
0
RFS
0
5
10
Years
U50B-High
U50B-Low
p=0.36
HR=0.72

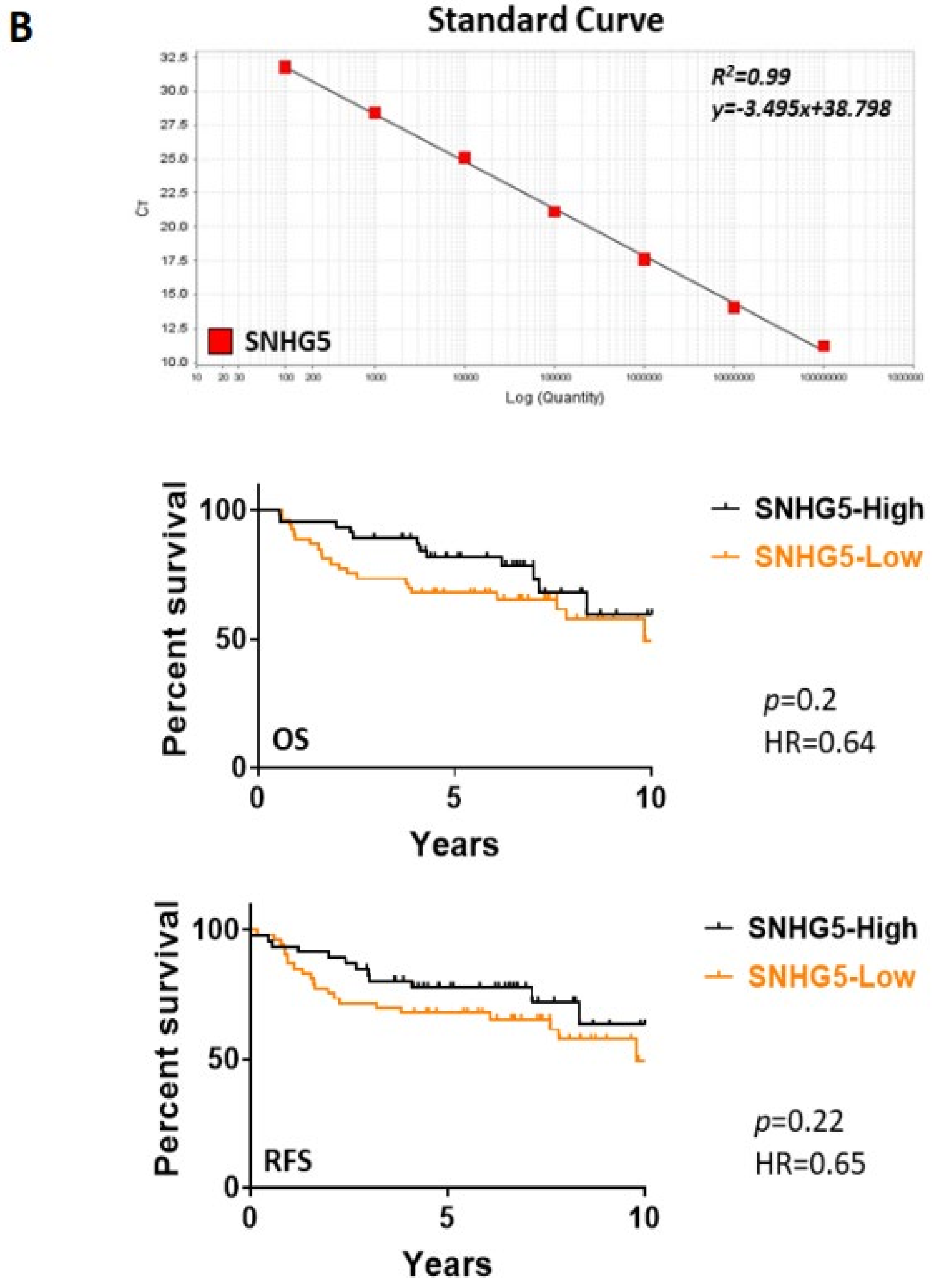

Figure S2. Survival analysis of U50B and SNHG5 in breast cancer patients. Absolute quantification of copy number of U50B and SNHG5. The standard curve of U50B (**A**) and SNHG5 (**B**). Ten-fold serial dilutions of plasmid templates were used. Kaplan-Meier curves for overall survival (OS) and relapse-free survival (RFS) of breast cancer patients. Median was used as the cutoff to separate U50B-high and U50B-low group or SNHG5-high and SNHG5-low group.

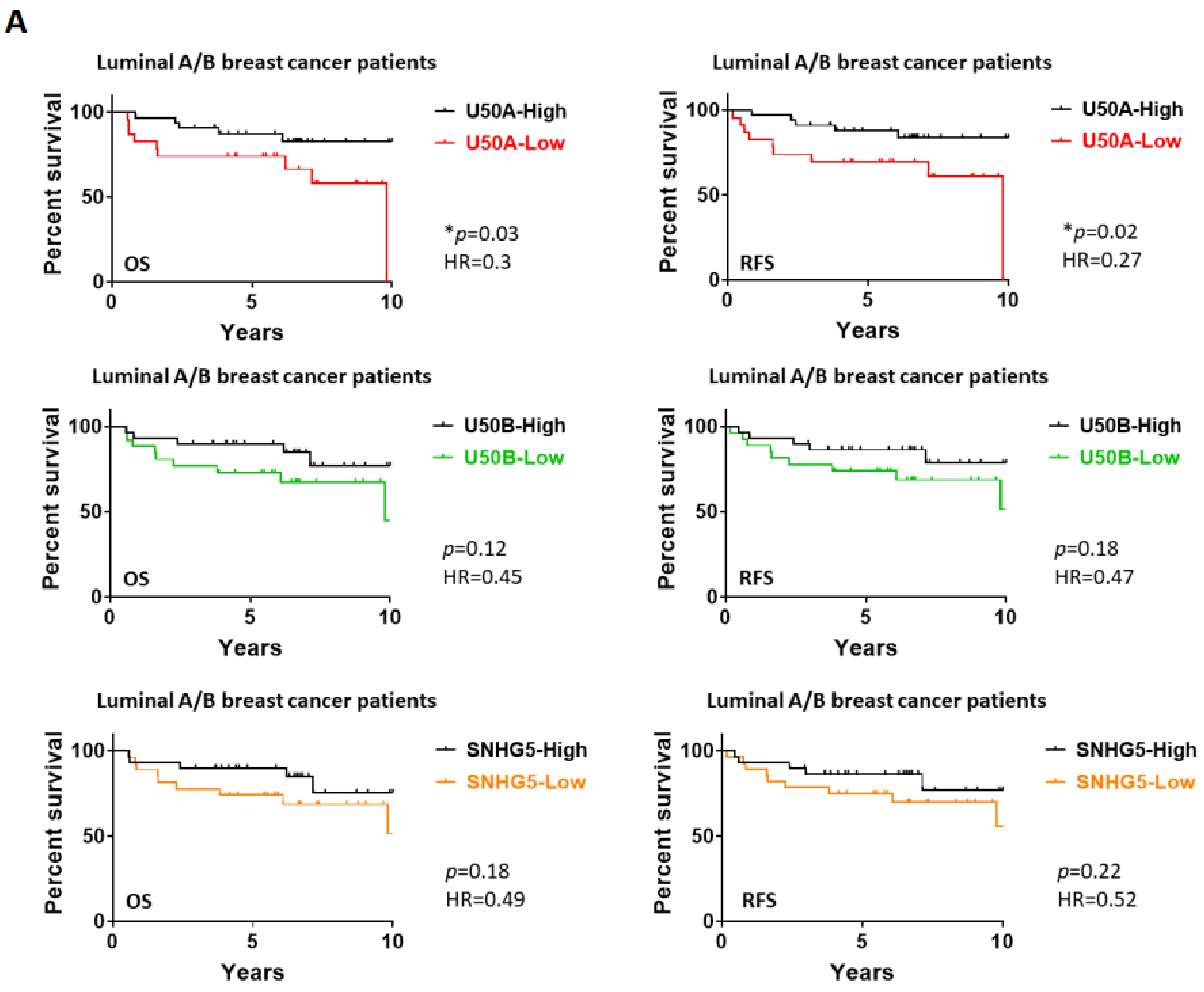
A
Luminal A/B breast cancer patients
Percent survival
100
50
0
OS
0
5
10
Years
U50A-High
U50A-Low
*p=0.03
HR=0.3
Luminal A/B breast cancer patients
Percent survival
RFS
Years
U50A-High
U50A-Low
*p=0.02
HR=0.27
Luminal A/B breast cancer patients
Percent survival
OS
Years
U50B-High
U50B-Low
p=0.12
HR=0.45
Luminal A/B breast cancer patients
Percent survival
RFS
Years
U50B-High
U50B-Low
p=0.18
HR=0.47
Luminal A/B breast cancer patients
Percent survival
OS
Years
SNHG5-High
SNHG5-Low
p=0.18
HR=0.49
Luminal A/B breast cancer patients
Percent survival
RFS
Years
SNHG5-High
SNHG5-Low
p=0.22
HR=0.52

B

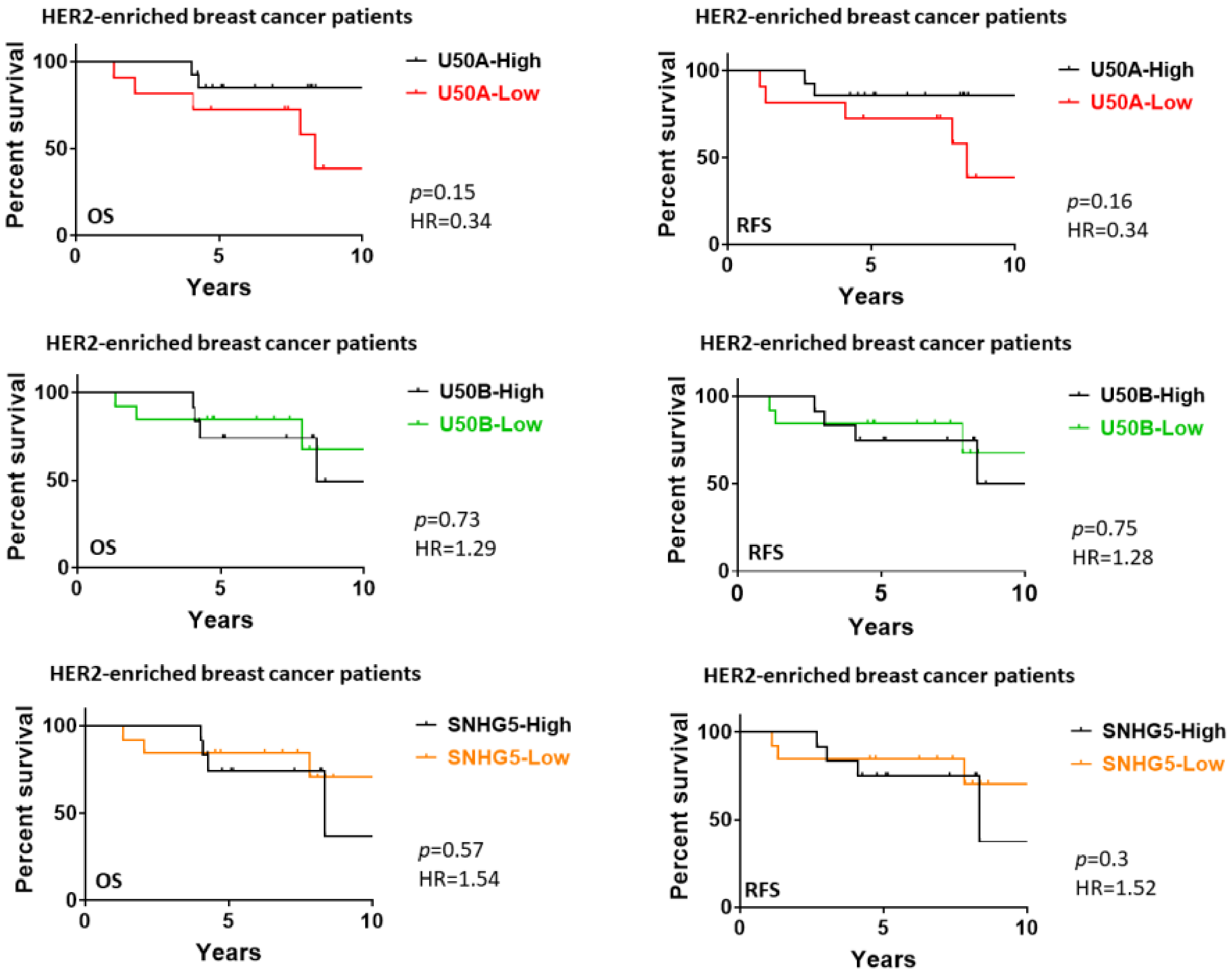
HER2-enriched breast cancer patients
Percent survival
100
50
0
OS
0
5
10
Years
U50A-High
U50A-Low
p=0.15
HR=0.34
HER2-enriched breast cancer patients
Percent survival
RFS
Years
U50A-High
U50A-Low
p=0.16
HR=0.34
HER2-enriched breast cancer patients
Percent survival
OS
Years
U50B-High
U50B-Low
p=0.73
HR=1.29
HER2-enriched breast cancer patients
Percent survival
RFS
Years
U50B-High
U50B-Low
p=0.75
HR=1.28
HER2-enriched breast cancer patients
Percent survival
OS
Years
SNHG5-High
SNHG5-Low
p=0.57
HR=1.54
HER2-enriched breast cancer patients
Percent survival
RFS
Years
SNHG5-High
SNHG5-Low
p=0.3
HR=1.52

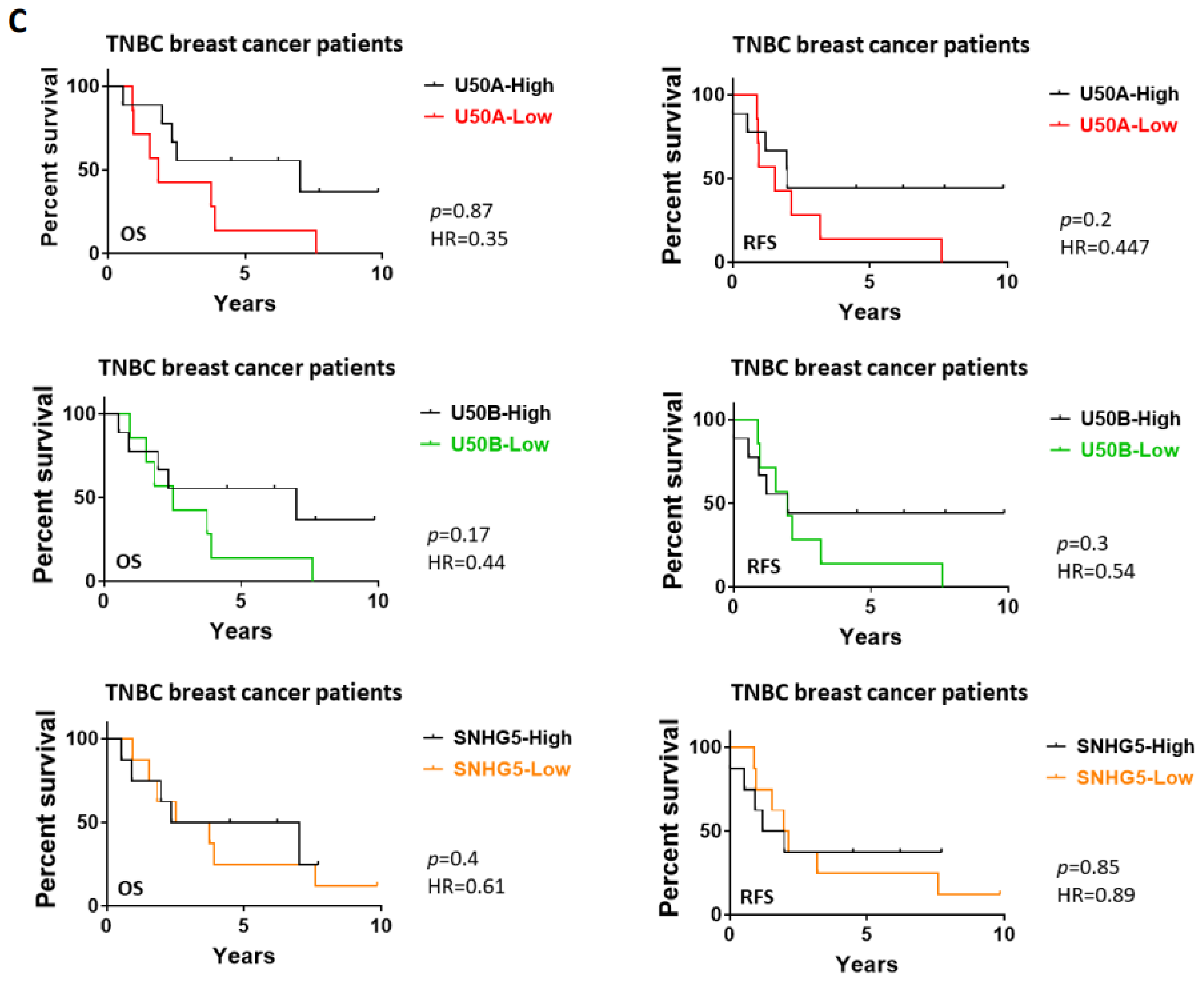

Figure S3. Survival analysis of U50A, U50B and SNHG5 in individual breast cancer subtypes. Overall survival and relapse-free survival, using U50A, U50B or SNHG5 as prognostic marker, in luminal A/B (**A**), HER2-enriched (**B**) and TNBC (**C**) breast cancer patients. * $p < 0.05$.

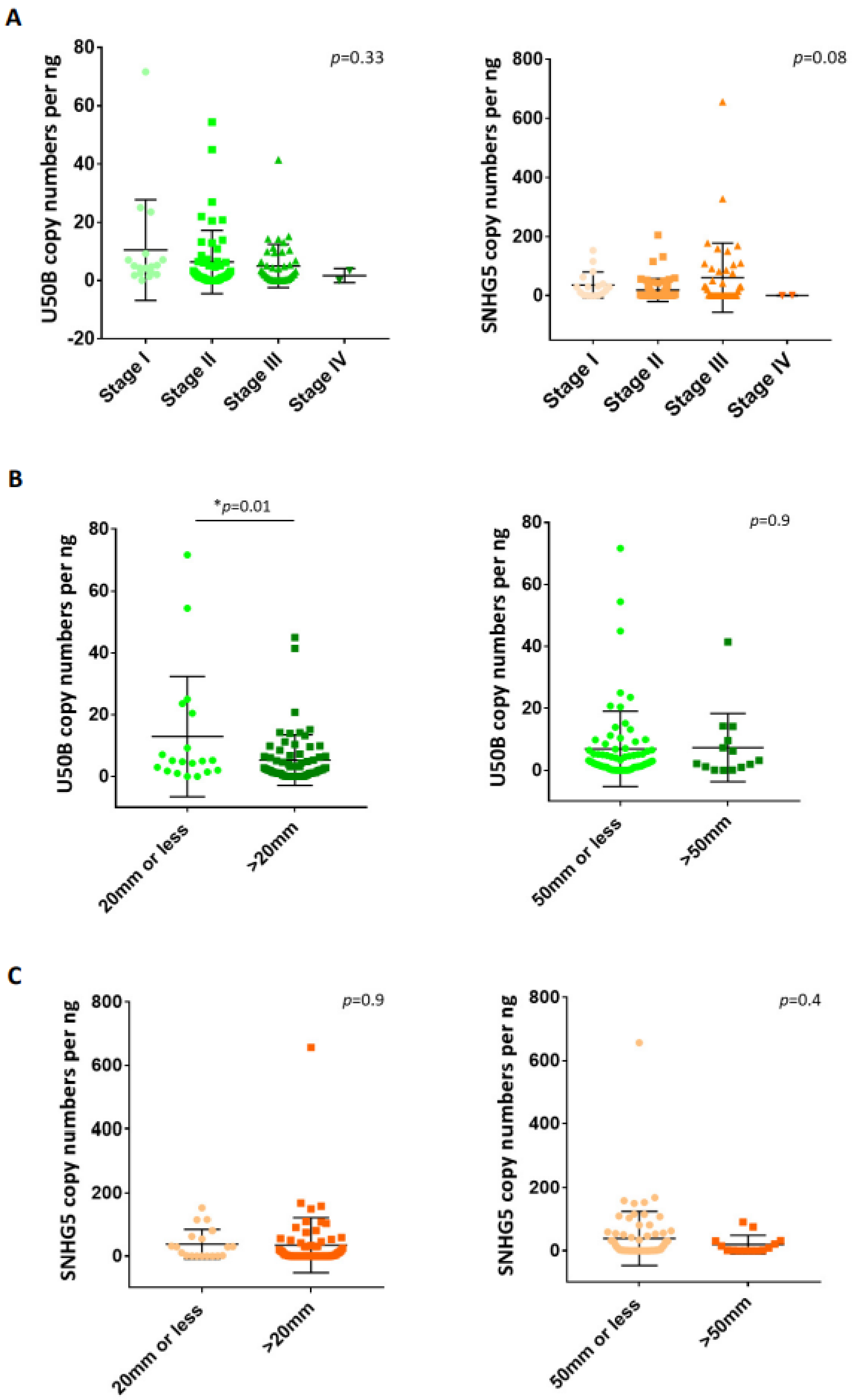
A
p=0.33
U50B copy numbers per ng
80
60
40
20
0
-20
Stage I
Stage II
Stage III
Stage IV
p=0.08
SNHG5 copy numbers per ng
800
600
400
200
0
Stage I
Stage II
Stage III
Stage IV
B
*p=0.01
U50B copy numbers per ng
20mm or less
>20mm
p=0.9
U50B copy numbers per ng
50mm or less
>50mm
C
p=0.9
SNHG5 copy numbers per ng
20mm or less
>20mm
p=0.4
SNHG5 copy numbers per ng
50mm or less
>50mm

Figure S4. Correlation between clinical characteristic and U50B, SNHG5 in breast cancer patients. Correlation between tumor stage (**A**), tumor size 20 mm (**B**), tumor size 50 mm (**C**) and U50B or SNHG5 in breast cancer patients. * $p < 0.05$.

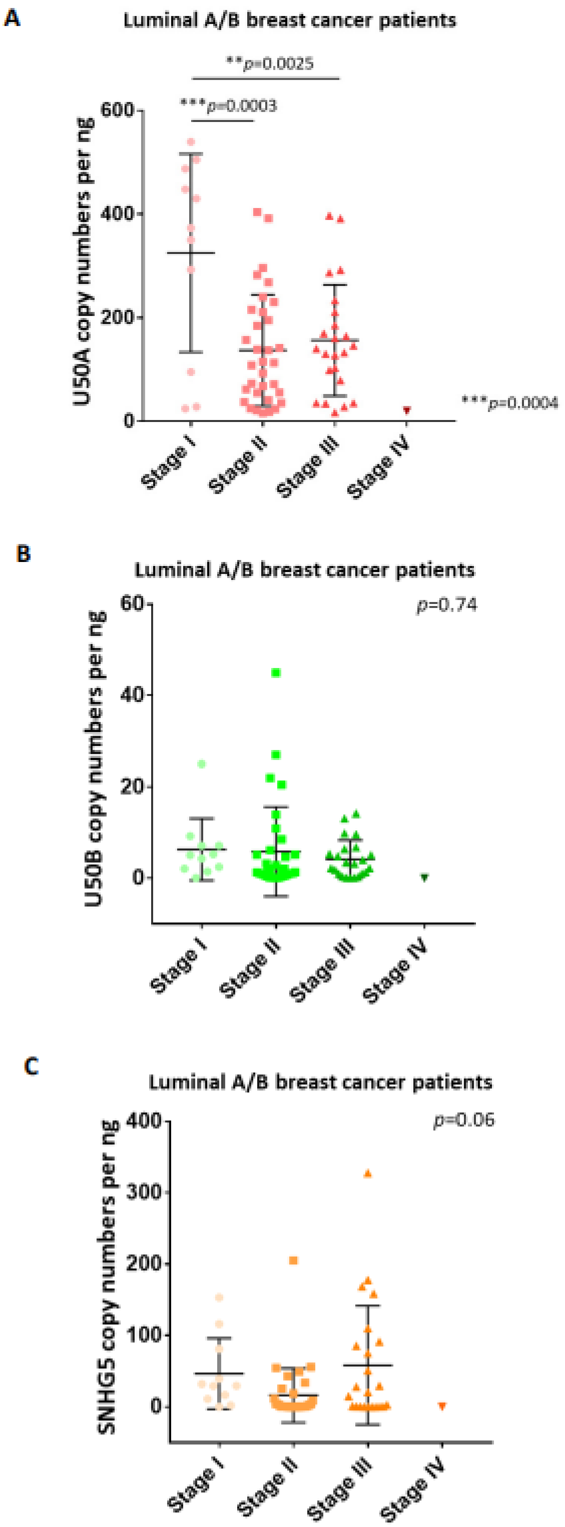

Figure S5. Correlation between clinical characteristic and U50A, U50B, SNHG5 in luminal A/B breast cancer patients. Correlation between tumor stage and U50A (**A**), U50B (**B**), SNHG5 (**C**) in luminal A/B breast cancer patients. *** $p < 0.001$.

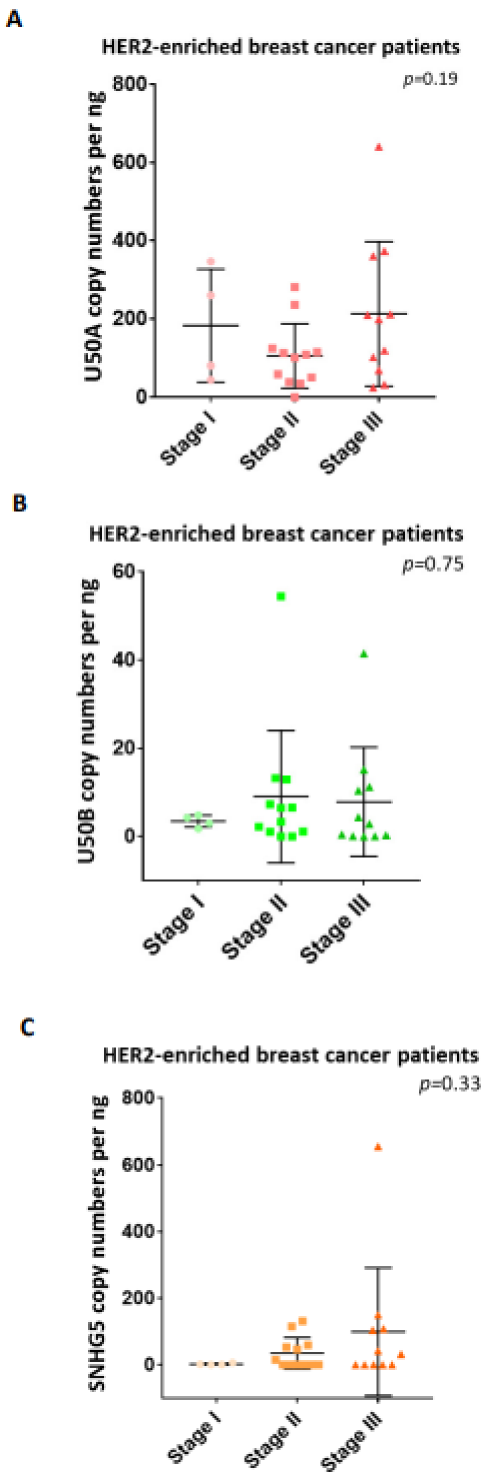

Figure S6. Correlation between clinical characteristic and U50A, U50B, SNHG5 in HER2-enriched breast cancer patients. Correlation between tumor stage and U50A (**A**), U50B (**B**), SNHG5 (**C**) in HER2-enriched breast cancer patients.

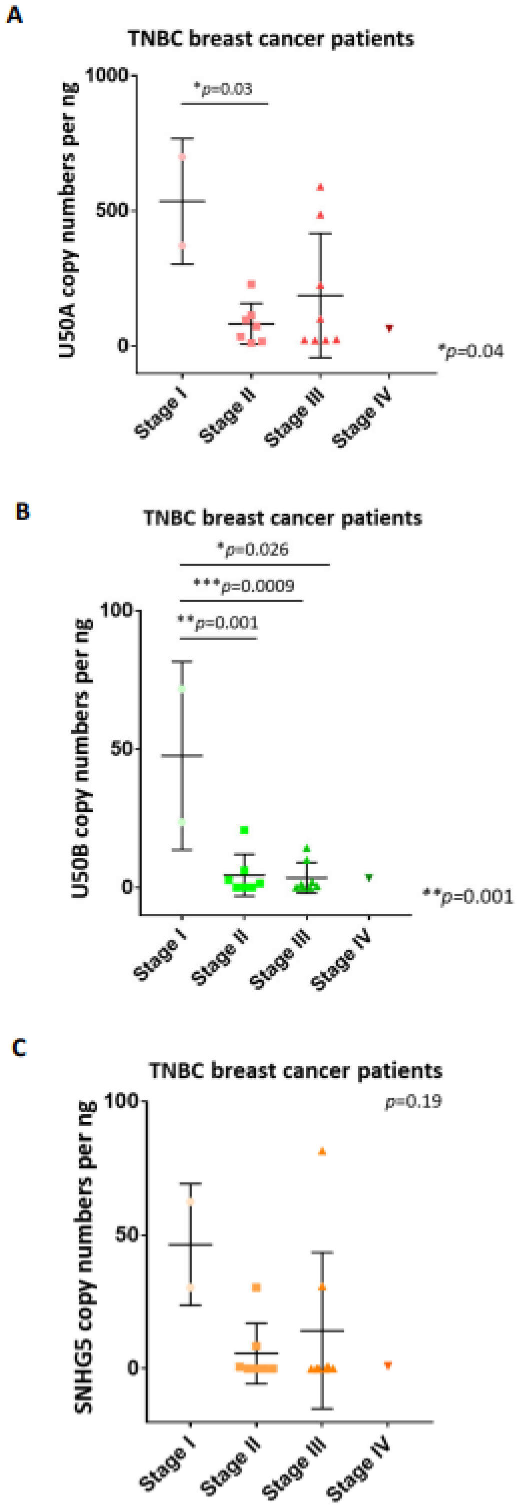

Figure S7. Correlation between clinical characteristic and U50A, U50B, SNHG5 in TNBC patients. Correlation between tumor stage and U50A (**A**), U50B (**B**), SNHG5 (**C**) in TNBC patients. * $p < 0.05$, ** $p < 0.01$, *** $p < 0.001$

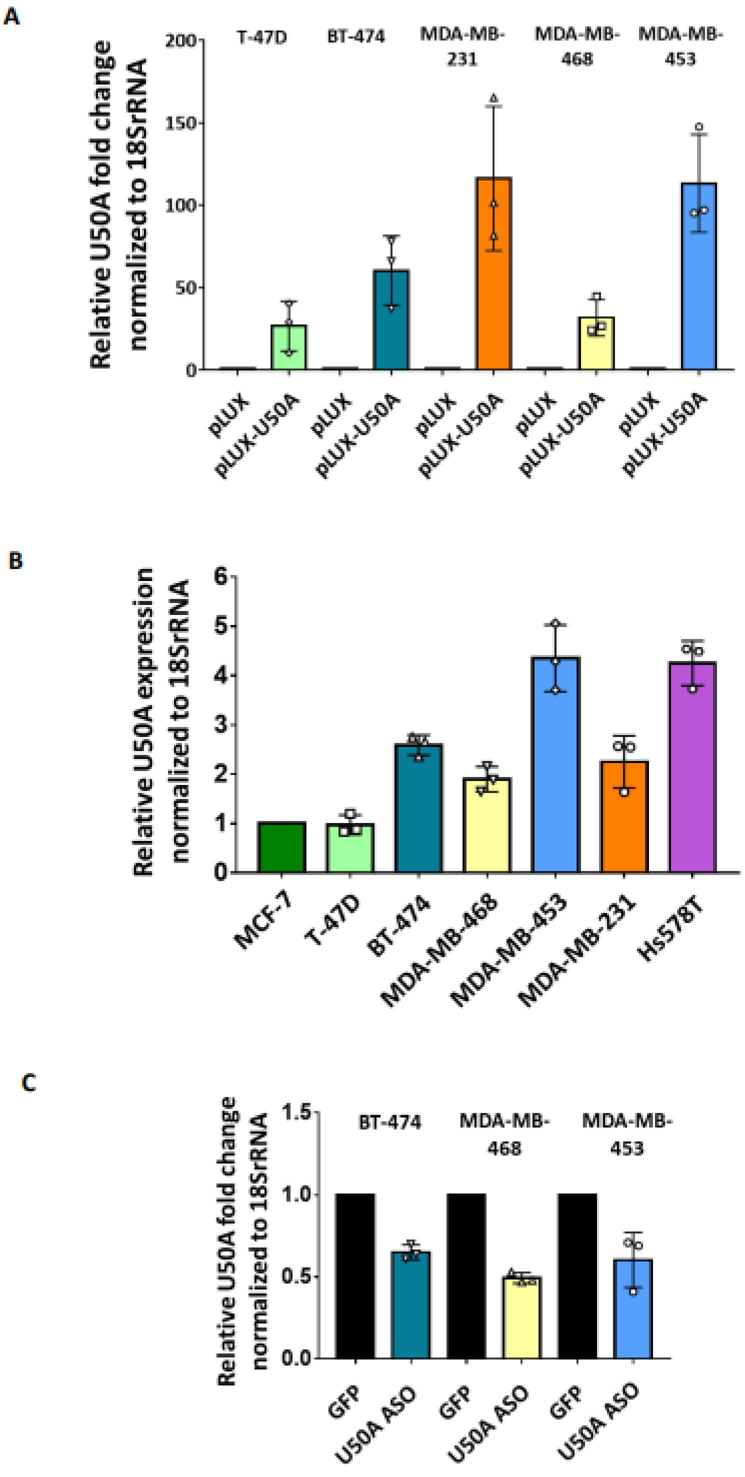

Figure S8. U50A expression in modulated groups. (**A**) U50A fold change in U50A-overexpressing breast cancer cell lines. (**B**) Relative endogenous U50A expression in several breast cancer cell lines. (**C**) U50A fold change in U50A-inhibiting breast cancer cell lines.

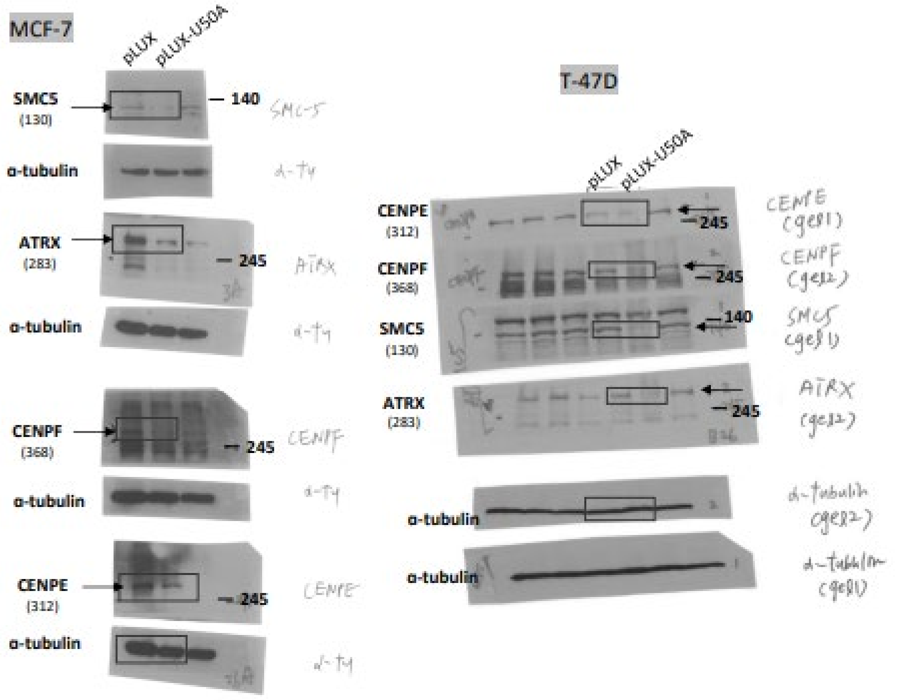
MCF-7
pLUX
pLUX-U50A
SMC5
(130)
140
α-tubulin
ATRX
(283)
245
α-tubulin
CENPF
(368)
245
α-tubulin
CENPE
(312)
245
α-tubulin
T-47D
pLUX
pLUX-U50A
CENPE
(312)
245
CENPF
(368)
245
SMC5
(130)
140
ATRX
(283)
245
α-tubulin
α-tubulin

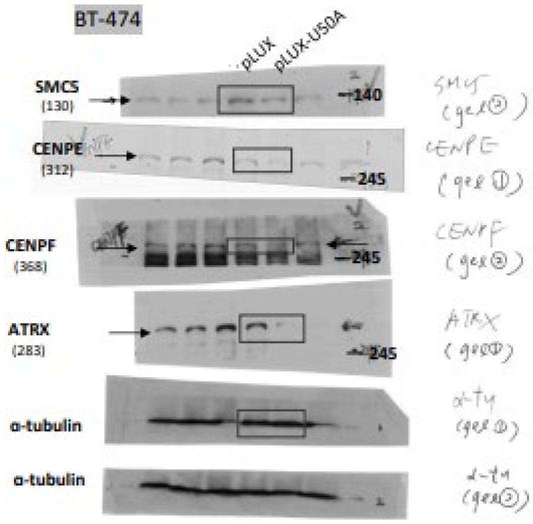
BT-474
pLUX
pLUX-U50A
SMC5
(130)
140
CENPE
(312)
245
CENPF
(368)
245
ATRX
(283)
245
α-tubulin
α-tubulin

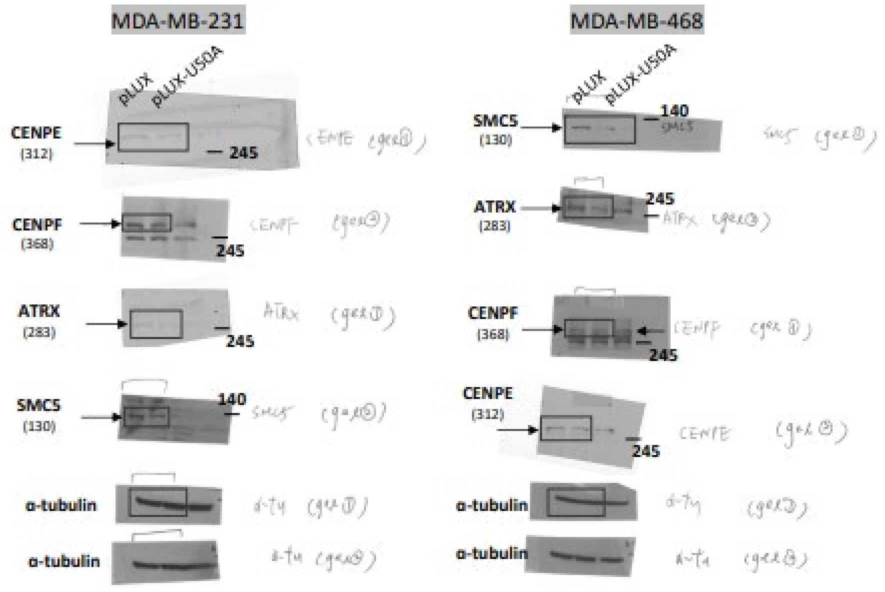
MDA-MB-231
pLUX
pLUX-U50A
CENPE
(312)
245
CENPF
(368)
245
ATRX
(283)
245
SMC5
(130)
140
α-tubulin
α-tubulin
MDA-MB-468
pLUX
pLUX-U50A
SMC5
(130)
140
ATRX
(283)
245
CENPF
(368)
245
CENPE
(312)
245
α-tubulin
α-tubulin

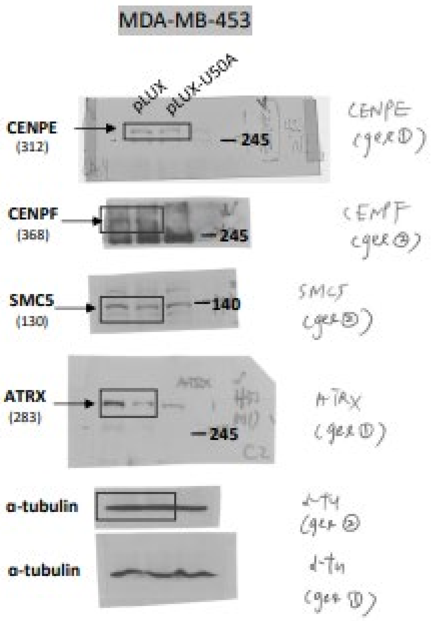
MDA-MB-453
pLUX
pLUX-U50A
CENPE
(312)
245
CENPF
(368)
245
SMC5
(130)
140
ATRX
(283)
245
α-tubulin
α-tubulin

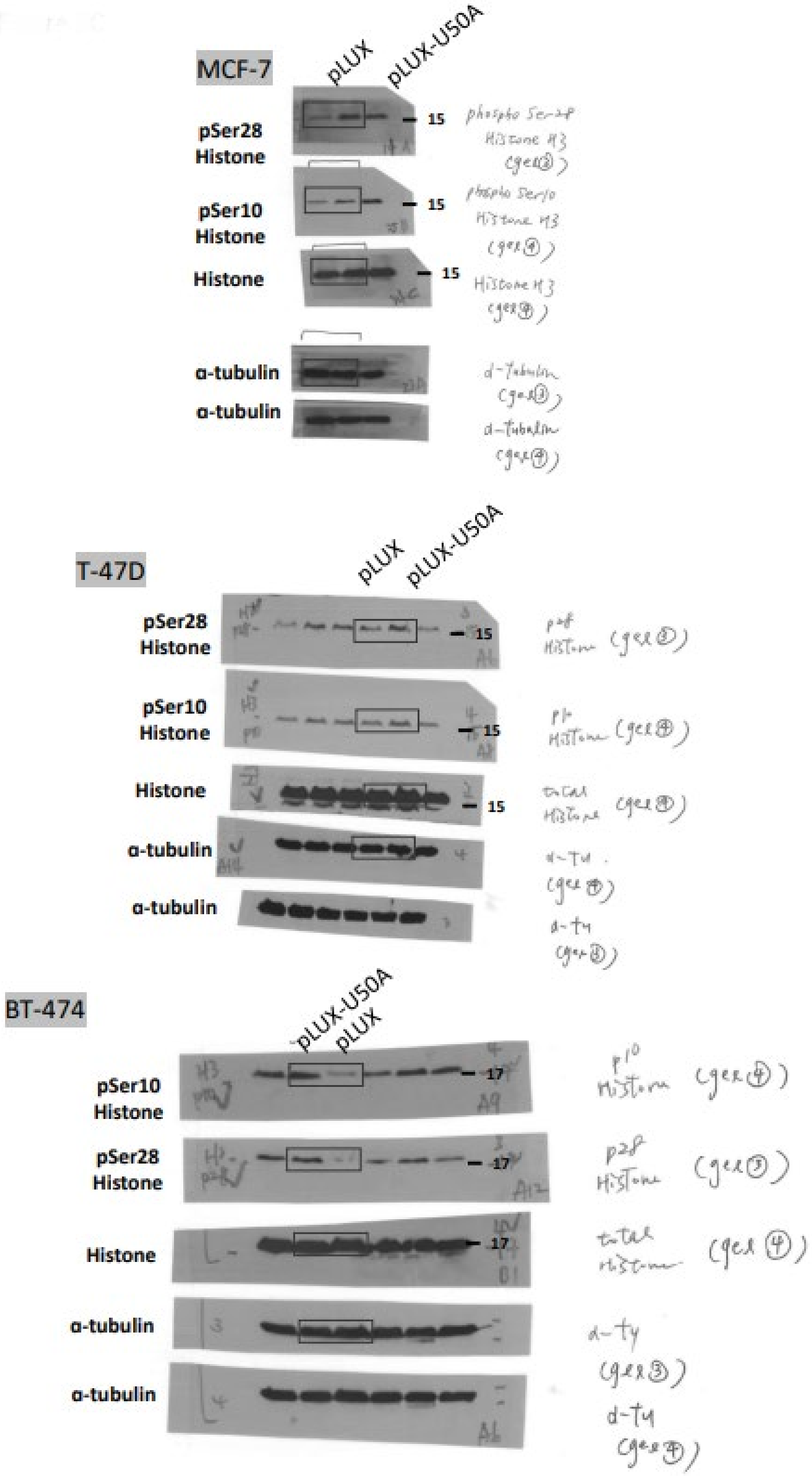
MCF-7
pLUX
pLUX-U50A
pSer28 Histone
pSer10 Histone
Histone
α-tubulin
α-tubulin
15
15
15
T-47D
pLUX
pLUX-U50A
pSer28 Histone
pSer10 Histone
Histone
α-tubulin
α-tubulin
15
15
15
BT-474
pLUX-U50A
pLUX
pSer10 Histone
pSer28 Histone
Histone
α-tubulin
α-tubulin
17
17
17

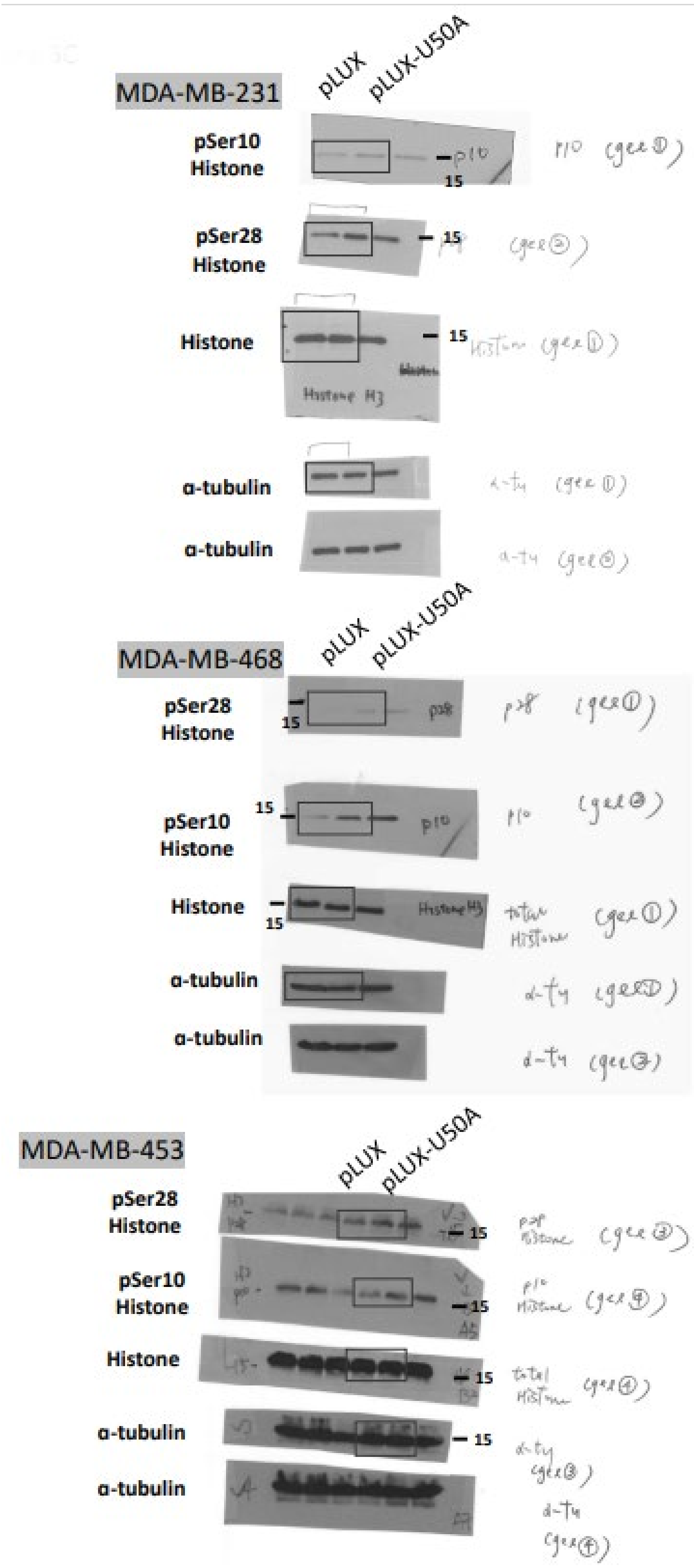
MDA-MB-231
pLUX
pLUX-U50A
pSer10 Histone
15
pSer28 Histone
15
Histone
15
α-tubulin
α-tubulin
MDA-MB-468
pLUX
pLUX-U50A
pSer28 Histone
15
pSer10 Histone
15
Histone
15
α-tubulin
α-tubulin
MDA-MB-453
pLUX
pLUX-U50A
pSer28 Histone
15
pSer10 Histone
15
Histone
15
α-tubulin
15
α-tubulin

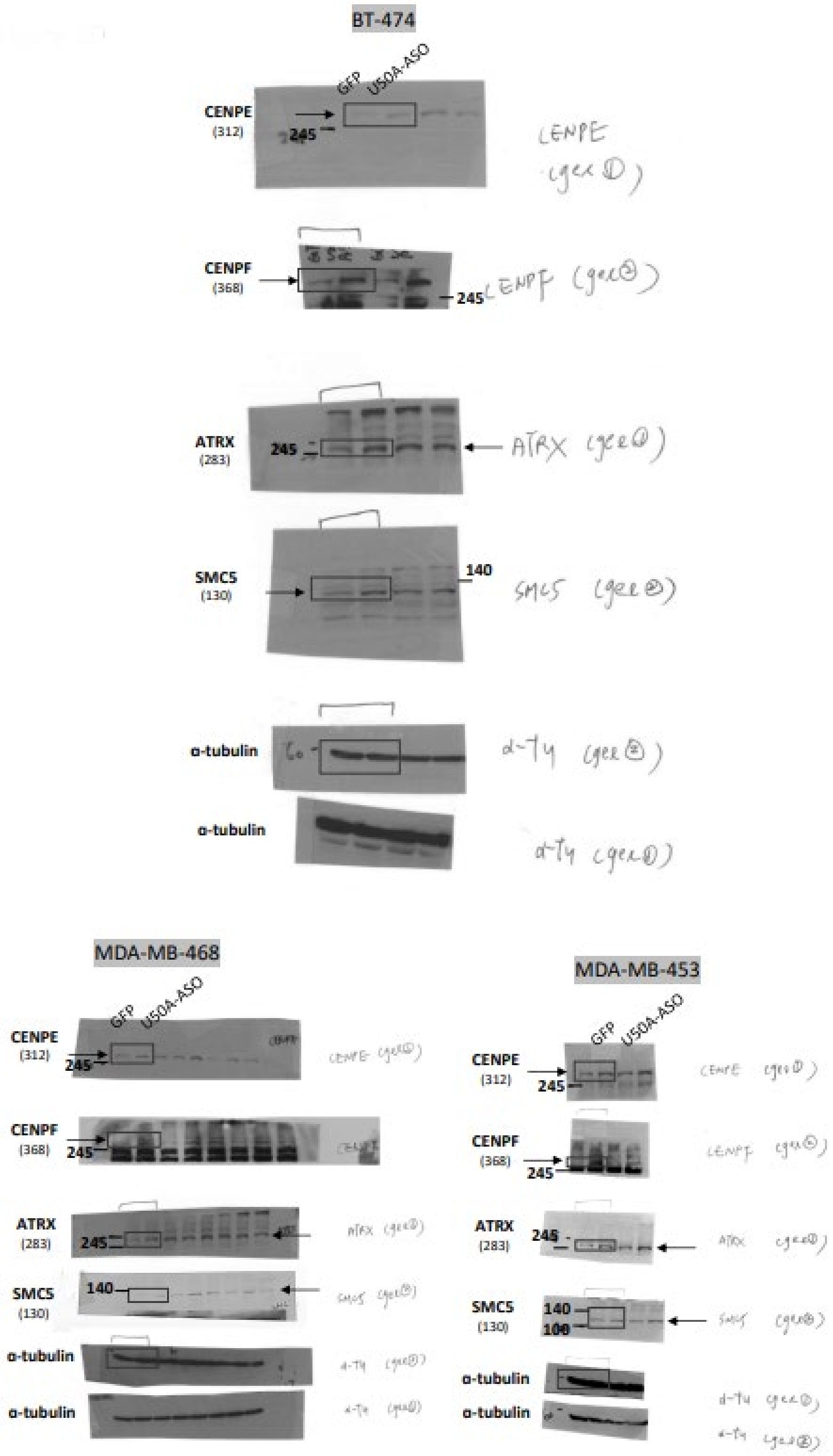
BT-474
GFP
U50A-ASO
CENPE
(312)
245
CENPF
(368)
245
ATRX
(283)
245
SMC5
(130)
140
α-tubulin
α-tubulin
MDA-MB-468
GFP
U50A-ASO
CENPE
(312)
245
CENPF
(368)
245
ATRX
(283)
245
SMC5
(130)
140
α-tubulin
α-tubulin
MDA-MB-453
GFP
U50A-ASO
CENPE
(312)
245
CENPF
(368)
245
ATRX
(283)
245
SMC5
(130)
140
100
α-tubulin
α-tubulin

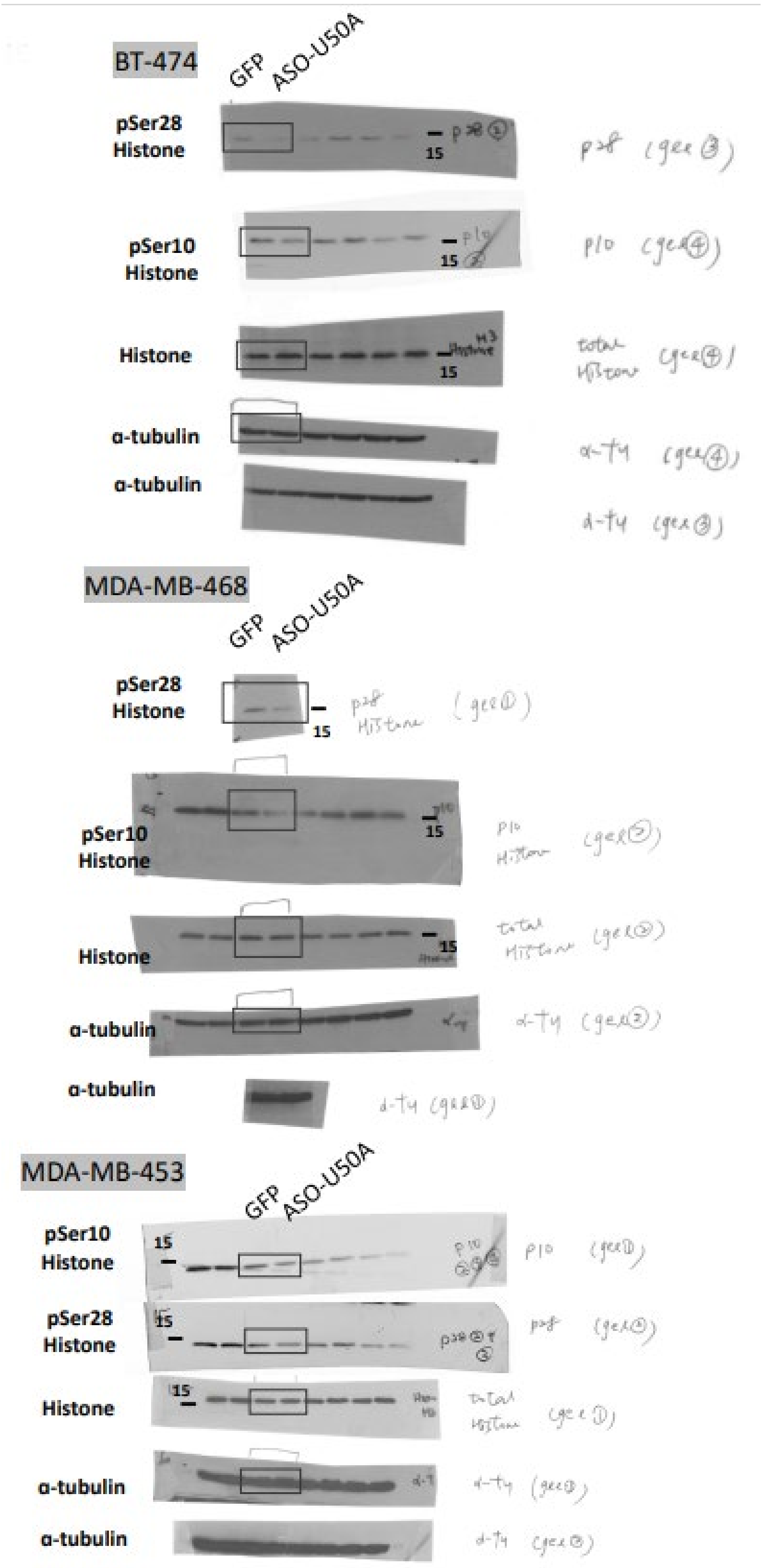

Figure S9. Uncropped WB for Figure 3B,C,D,E.

Table S1. Survival analysis of U50A, U50B and SNHG5 in breast cancer subtypes (number of patients).

Marker	Luminal A/B (68)				HER2-enriched (28)				Triple-negative (18)			
	OS		RFS		OS		RFS		OS		RFS	
**U50A**	*p*	HR	*p*	HR	*p*	HR	*p*	HR	*p*	HR	*p*	HR
	0.03 *	0.3 #	0.02 *	0.27 #	0.15	0.34 #	0.16	0.34 #	0.87	0.35#	0.2	0.45 #
	OS		RFS		OS		RFS		OS		RFS	
**U50B**	*p*	HR	*p*	HR	*p*	HR	*p*	HR	*p*	HR	*p*	HR
	0.12	0.45#	0.18	0.47#	0.73	1.29	0.75	1.28	0.17	0.44 #	0.3	0.54
	OS		RFS		OS		RFS		OS		RFS	
**SNHG5**	*p*	HR	*p*	HR	*p*	HR	*p*	HR	*p*	HR	*p*	HR
	0.18	0.49 #	0.22	0.52	0.57	1.54	0.3	1.52	0.4	0.61	0.85	0.89

Survival analysis was analyzed by Log-rank test. # Hazard ratio (HR) <0.5. * $p < 0.05$. Original data from Figure S2.

Table S2. Correlation between copy numbers of U50A, U50B or SNHG5 with pathological stages in breast cancer subtypes.

Luminal A/B	Stage (Mean ± S.D.)				
**Marker**	**I**	**II**	**III**	**IV**	***p***
U50A	325 ± 191.3	137.2 ± 107.4	156.6 ± 107.3	20 ± 0	*** 0.0004
U50B	6.28 ± 6.8	5.81 ± 9.8	4.17 ± 4.15	0.01 ± 0	0.74
SNHG5	46.45 ± 49.57	16.09 ± 38.07	58.42 ± 83.23	0.01 ± 0	0.06
HER2-enriched Marker					
U50A	182.6 ± 144.2	104.8 ± 81.97	212.7 ± 184.6	-	0.19
U50B	3.46 ± 1.34	9.05 ± 15	7.84 ± 12.38	-	0.75
SNHG5	2.18 ± 2.81	35.08 ± 47.25	99.35 ± 192.3	-	0.33
TNBC Marker					
U50A	536 ± 232.1	82.33 ± 75.56	187.1 ± 231	64.4 ± 0	* 0.04
U50B	47.63 ± 34.02	4.46 ± 7.53	3.49 ± 5.48	3.4 ± 0	** 0.0012
SNHG5	46.51 ± 22.73	5.67 ± 11.35	14.23 ± 29.3	0.97 ± 0	0.19

S.D.: Standard error of the mean, * $p < 0.05$, ** $p < 0.01$, *** $p < 0.001$. Stage according to the recommendations of the American Cancer Society, Use One-way ANOVA, Tukey's multiple comparisons test, original data from Figures S4–S6.

Table S3. Multivariate Analysis of breast cancer prognostic markers.

Variable	Overall survival (OS)			Relapse-free survival (RFS)		
	p	**95% Confidence Interval**	**Hazard Ratio**	***p***	**95% Confidence Interval**	**Hazard Ratio**
T	0.01 *	1.293–6.475	2.894	0.008 **	1.333-6.745	2.999
N	0.78	0.210–3.227	0.823	0.741	0.203-3.103	0.795
Stage	0.024 *	1.211–16.10	4.416	0.028 *	1.170-15.721	4.288
U50A	0.098	0.239–1.128	0.519	0.063	0.222-1.039	0.480

Based on Cox proportional hazards model. * $p < 0.05$, ** $p < 0.01$
